# Gluconic acid improves performance of newly weaned piglets associated with alterations in gut microbiome and fermentation

**DOI:** 10.1186/s40813-023-00305-1

**Published:** 2023-04-05

**Authors:** Joris Michiels, Damien Truffin, Maryam Majdeddin, Mario Van Poucke, Elout Van Liefferinge, Noémie Van Noten, Mario Vandaele, Céline Van Kerschaver, Jeroen Degroote, Luc Peelman, Pierre Linder

**Affiliations:** 1 Laboratory for Animal Nutrition and Animal Product Quality, Department of Animal Sciences and Aquatic Ecology, Ghent University, Campus Coupure, Coupure Links 653, 9000 Ghent, Belgium; 2 Roquette Frères, 1 rue de La Haute Loge, 62136 Lestrem, France; 3 Department of Veterinary and Biosciences, Ghent University, Heidestraat 19, 9820 Merelbeke, Belgium

**Keywords:** Piglet, Weaning, Gluconic acid, Butyrate, *Lactobacillus amylovorus*, *Megasphaera elsdenii*

## Abstract

**Background:**

Weaning is a critical phase in the pigs’ life and gut health might be compromised. Gluconic acid was shown to be poorly absorbed but readily fermented to butyrate in the gut which in turn can improve gut function. Hence, a total of 144 weaning pigs were fed the experimental diets for 42 days. Three treatments were replicated in 8 pens with 6 piglets each: control; low dietary dose of gluconic acid, 9 g/kg; and high dietary dose of gluconic acid, 18 g/kg. After 21 days, one piglet from each pen was sampled for blood haematology and biochemistry, fore- and hindgut digesta characteristics and microbiota, and distal small intestinal histo-morphological indices and gene expression.

**Results:**

Feeding gluconic acid enhanced performance in period d 0–14 post-weaning, in particular feed intake was increased (*P* = 0.028), though the high dose did not show benefits over the low dose. Regarding d 0–42, feed intake was elevated (*P* = 0.026). At d 21, piglets fed 18 g/kg gluconic acid showed a trend for lower number of total white blood cells (*P* = 0.060), caused by particularly lower numbers of lymphocytes as compared to control (*P* = 0.028). Highly reduced plasma urea was found for groups fed gluconic acid, it amounted to 2.6 and 2.6 mmol/L for the 9 and 18 g/kg level, respectively, as compared to 3.8 mmol/L in control (*P* = 0.003). Feeding gluconic acid promoted the relative abundance of lactic-acid-producing and acid-utilizing bacteria. In distal small intestine, *Lactobacillus amylovorus* increased substantially from 11.3 to 82.6% for control and gluconic acid 18 g/kg, respectively (*P* < 0.05). In mid-colon, the butyrate producers *Faecalibacterium prausnitzii* (*P* > 0.05) and *Megasphaera elsdenii* (*P* < 0.05) showed highest abundance in gluconic acid 18 g/kg. Consequently, in caecum and mid-colon, increased relative molar percentage of butyrate were found, e.g., 10.0, 12.9 et 14.7% in caecum for gluconic acid at 0, 9, and 18 g/kg, respectively (*P* = 0.046). Elevated mRNA anti-inflammatory cytokine and survival signalling levels in distal small intestinal mucosa were found by feeding gluconic acid which might be mediated by butyrate.

**Conclusions:**

Gluconic acid may have potential to alleviate the postweaning growth-check in pigs by altering microbiota composition and fermentation in the gut.

**Supplementary Information:**

The online version contains supplementary material available at 10.1186/s40813-023-00305-1.

## Background

Piglets weaned at 3 to 4 weeks of age are exposed to nutritional, environmental, and social stresses leading to low feed intake, reduced weight gain, nutrient malabsorption, and increased occurrence of diarrhoea [1]. Antibiotics and trace elements like Cu and ZnO have been used widely to limit the impact of the postweaning period on animal health. Nevertheless, feeding these antimicrobials to farm animals may be responsible for the spreading of bacteria that are resistant to such antimicrobials [2, 3]. Yet, the quest for valuable nutritional and management alternatives continues.

Previously, it was shown that gluconic acid (C_6_H_12_O_7_, CAS 526-95-4) is poorly absorbed in the gastrointestinal tract of the rat [4], but prone to fermentation stimulating butyrate production. Tsukahara et al. [5] outlined in an in vitro porcine caecal model that gluconic acid is slowly fermented by lactic acid bacteria such as *Lactobacillus reuteri* and *Lactobacillus mucosae*, allowing the lactate and acetate that were produced to be converted to butyrate by acid-utilizing bacteria, such as *Megasphaera elsdenii* and *Mitsuokella multacida*. Accordingly, total short-chain fatty acids (SCFA), acetate, propionate, butyrate, acetate + butyrate to propionate ratio were linearly increased by gluconic acid, whereas ammonia was reduced, in 24 h in vitro porcine caecal fermentations [6]. In an in vitro comparison of several prebiotics, gluconic acid yielded the highest butyrate molar ratio after 12 h (29.4%) with a particular reduction of propionate, though no reduction in *Salmonella* counts nor any effects on other bacteria were seen [7]. To recall, butyrate is the major energy source of the epithelial cells of the large intestine, and hence fosters cell proliferation, mucus production and water and mineral absorption. Moreover, butyrate operates as a signal metabolite in the homeostasis of epithelial cells, regulating the balance between proliferation, differentiation, and apoptosis, control of intestinal barrier function, and control of cytokine production, amongst other effects, in the small intestine even though butyrate is produced in the colon, presumably indirectly by neurohormonal mechanisms [8, 9].

Even though feeding gluconic acid to piglets might be an appealing approach to sustain gut function and alleviate the postweaning growth-check in piglets, few studies have reported the effect of gluconic acid in weaned piglets. Biagi et al. [6] showed that 3 and 6 g/kg gluconic acid in the diet increased daily gain of piglets opposite to 12 g/kg gluconic acid, while no effects on feed intake and gain-to-feed ratio, nor on intestinal counts of bacteria or histo-morphological indices were found. However, gluconic acid tended to increase SCFA in jejunal contents.

For the current study, it was hypothesized that dietary gluconic acid, and up to higher levels compared to the study of Biagi et al. [6], could be fermented to deliver enhanced butyrate levels in the gastro-intestinal tract of weaning piglets, and accordingly improve performance and health. Hence, a total of 144 weaning pigs were fed the experimental diets for 42 days. Three treatments were replicated in 8 pens with 6 piglets each: control; low dietary dose of gluconic acid, 9 g/kg; and high dietary dose of gluconic acid, 18 g/kg. After 21 days, one piglet from each pen was sampled for blood haematology and biochemistry, fore- and hindgut digesta characteristics and microbiota, and distal small intestinal histo-morphological indices and gene expression.

## Results

### Animal performances and health

No major health issues occurred during the experiment, apart from some piglets showing continued swollen joints, coughing, and/or general runting. Two piglets from control were culled and one piglet succumbed suddenly (treatment gluconic acid at 9 g/kg). Limited individual antibiotic treatments (1.8% of animal days) were executed, mostly amoxicillin, and this was not different across treatments. Faecal consistency scores peaked between d 4 and 8 postweaning indicative for softer and more liquid excreta, after which a sharp drop was observed, followed by a transient smaller increase after the dietary switch at d 14, though with limited differences between treatments (Additional file 1). In period d 4–7, nearly all pens had piglets with diarrhoea with incidence exceeding 15%, though again limited differences between treatments were found (Additional file 1). In contrast, feeding gluconic acid had a prominent effect on performance in the pre-starter period: a trend for higher final BW (body weight) (at d 14) (*P* = 0.061) and ADG (average daily gain) (*P* = 0.069), increased feed intake (*P* = 0.028), concomitant with a trend for improved F:G (feed-to-gain ratio) (*P* = 0.055) (Table 1). ADFI (average daily feed intake) for pre-starter diet was higher for gluconic acid at 9 g/kg as compared to control (*P* < 0.05), and this difference was yet obvious on d 5 postweaning (*P* < 0.05, Additional file 2). Further, a trend for lower W:F (water-to-feed ratio) was found (*P* = 0.095), suggesting lower water consumption relative to feed intake when gluconic acid was in the diet. Performances in starter period were in line with pre-starter period, though significance was only found for feed intake in period d 28–42 (ADFI of piglets fed gluconic acid was higher as compared to control, *P* < 0.05), even though body weight at the end seems substantially higher in gluconic acid fed pigs (+ 1.9 and 0.9 kg as compared to control, for treatments gluconic acid at 9 and 18 g/kg, respectively, *P* > 0.05). Overall (d 0–42), it can be observed that feed intake was increased with gluconic acid in the diet as compared to control (*P* = 0.026). In line with performance effects, some differences in apparent ileal digestibility of major nutrients were observed (no statistics, based on pooled samples per treatment). *In concreto*, treatment gluconic acid at 18 g/kg showed 0.046, 0.044 and 0.037 points higher dry matter, organic matter, and crude protein digestibility, respectively, as compared to control, whereas results for control and gluconic acid at 9 g/kg were similar (Table 2).

**Table 1:** Effect of diet on performance indices of piglets fed the experimental diets (n = 8)^ab^

Item	Gluconic acid (g/kg)			SEM	*P*
	0	9	18		
*d 0–14*					
Initial BW (kg)	8.16	8.18	8.17	0.02	0.937
Final BW (kg)	9.49	10.07	9.9	0.11	0.061
ADG (g/d)	95	135	124	8	0.069
ADFI (g/d)	181^b^	224^a^	205^ab^	7	0.028
F:G (g/g)	1.93	1.69	1.68	0.05	0.055
ADWI (mL/d)	596	675	627	21	0.355
W:F (mL/g)	3.3	2.9	3.1	0.1	0.095
*d 14–28*					
Final BW (kg)	14.2	14.9	14.6	0.2	0.22
ADG (g/d)	323	337	331	7	0.756
ADFI (g/d)	429	474	469	10	0.146
F:G (g/g)	1.38	1.42	1.43	0.02	0.545
ADWI (mL/d)	1171	1298	1245	51	0.643
W:F (mL/g)	2.6	2.6	2.7	0.1	0.911
*d 28–42*					
Final BW (kg)	21.3	22.6	22.2	0.3	0.157
ADG (g/d)	509	552	543	13	0.389
ADFI (g/d)	726^b^	799^a^	794^a^	14	0.039
F:G (g/g)	1.43	1.44	1.46	0.01	0.58
ADWI (mL/d)	1843	2061	2078	86	0.507
W:F (mL/g)	2.5	2.6	2.7	0.1	0.831
*d 0–42*					
ADG (g/d)	309	341	332	7	0.147
ADFI (g/d)	424^b^	481^a^	471^a^	10	0.026
F:G (g/g)	1.47	1.46	1.48	0.01	0.834
ADWI (mL/d)	1157	1303	1245	49	0.532
W:F (mL/g)	2.7	2.7	2.7	0.1	0.955

^a^Body weight, BW; average daily gain, ADG; average daily feed intake, ADFI; feed-to-gain ratio, F:G; average daily water intake, ADWI; and water-to-feed ratio, W:F

^b^Means within row without common superscript are significantly different, P < 0.05

**Table 2:** Effect of diet on apparent ileal digestibility of nutrients in piglets fed the experimental diets^a^

Item	Gluconic acid (g/kg)		
	0	9	18
Dry matter	0.613	0.617	0.659
Organic matter	0.637	0.642	0.681
Crude protein (Nx6.25)	0.686	0.674	0.723

^a^Contents of the last half meter of the small intestine were collected from piglets at d 21, and subsequently pooled per treatment, freeze dried and used for measuring digestibility by the indicator method (marker: 4 mol/L HCl insoluble ash)

### Blood haematology and biochemistry

Haematological indices of piglets sampled on d 21 did not reveal any effect on red blood cells, however, dose-dependent effects on white blood cell populations were seen. Piglets fed gluconic acid at 18 g/kg showed lower number of total white blood cells (*P* < 0.05, Table 3), caused by particularly lower numbers of lymphocytes, as compared to control (*P* < 0.05). A trend towards a decrease of numbers of neutrophils in gluconic acid fed groups was found (*P* = 0.060). Mean platelet volume was affected by treatment with higher values when diets were supplemented with gluconic acid (*P* = 0.046). Among the blood biochemical indices, highly reduced plasma urea was found for both groups fed gluconic acid (P = 0.003), it amounted to 3.8, 2.6 and 2.6 mmol/L for control, gluconic acid at 9 g/kg, and gluconic acid at 18 g/kg, respectively.

**Table 3:** Effect of diet on blood indices in piglets fed the experimental diets and sampled on d 21 (n = 8)^ab^

Item	Gluconic acid (g/kg)			SEM	*P*
	0	9	18		
Haematology on whole blood					
Red blood cells (10^12^/L)	6.3	6.2	6.2	0.1	0.872
Haematocrit (%)	35.9	37.3	35.7	0.6	0.478
Haemoglobin (g/dL)	10	10.6	10.2	0.2	0.457
Mean corpuscular volume (fL)	56.8	61.8	57.5	1	0.092
Mean corpuscular haemoglobin (pg)	15.8	17	16.5	0.2	0.116
Mean corpuscular haemoglobin concentration (g/dL)	27.9	27.7	28.6	0.2	0.204
Reticulocytes (%)	1.53	1.43	1.79	0.16	0.66
White blood cells (K/μL)	94	88.1	110.1	9.7	0.651
Lymphocytes (%)	28.8^a^	26.5^a^	18.2^b^	1.5	0.005
Monocytes (%)	47.8	46.7	45.7	1.3	0.817
Neutrophils (%)	8.1	9.5	9.4	0.4	0.283
Eosinophils (%)	42.7	41.6	42.5	1.5	0.955
Basophils (%)	1.3	2.1	2.2	0.2	0.115
Lymphocytes (K/μL)	0.19	0.17	0.19	0.02	0.964
Monocytes (K/μL)	13.2^a^	12.3^ab^	8.3^b^	0.8	0.028
Neutrophils (K/μL)	2.3	2.6	1.7	0.2	0.173
Eosinophils (K/μL)	11.3	11	7.8	0.7	0.06
Basophils (K/μL)	0.32	0.5	0.4	0.04	0.24
Thrombocytes (K/μL)	0.053	0.049	0.03	0.006	0.229
Mean platelet volume (fL)	400	310	397	25	0.256
Biochemical indices	11.2	11.5	12.1	0.2	0.046
Creatinine (μmol/L)	83.5	79	77.8	2.1	0.504
NEFA ^c^ (mmol/L)	0.052	0.069	0.068	0.007	0.521
Total protein (g/L)	46.7	45.1	44.4	0.6	0.247
Urea (mmol/L)	3.8^a^	2.6^b^	2.6^b^	0.2	0.003

^a^Piglets were sampled on d 21

^b^Means within row without common superscript are significantly different, P < 0.05

^c^Non-esterified fatty acids

### Characteristics of digesta and metagenomic analysis

**Fig. 1:**
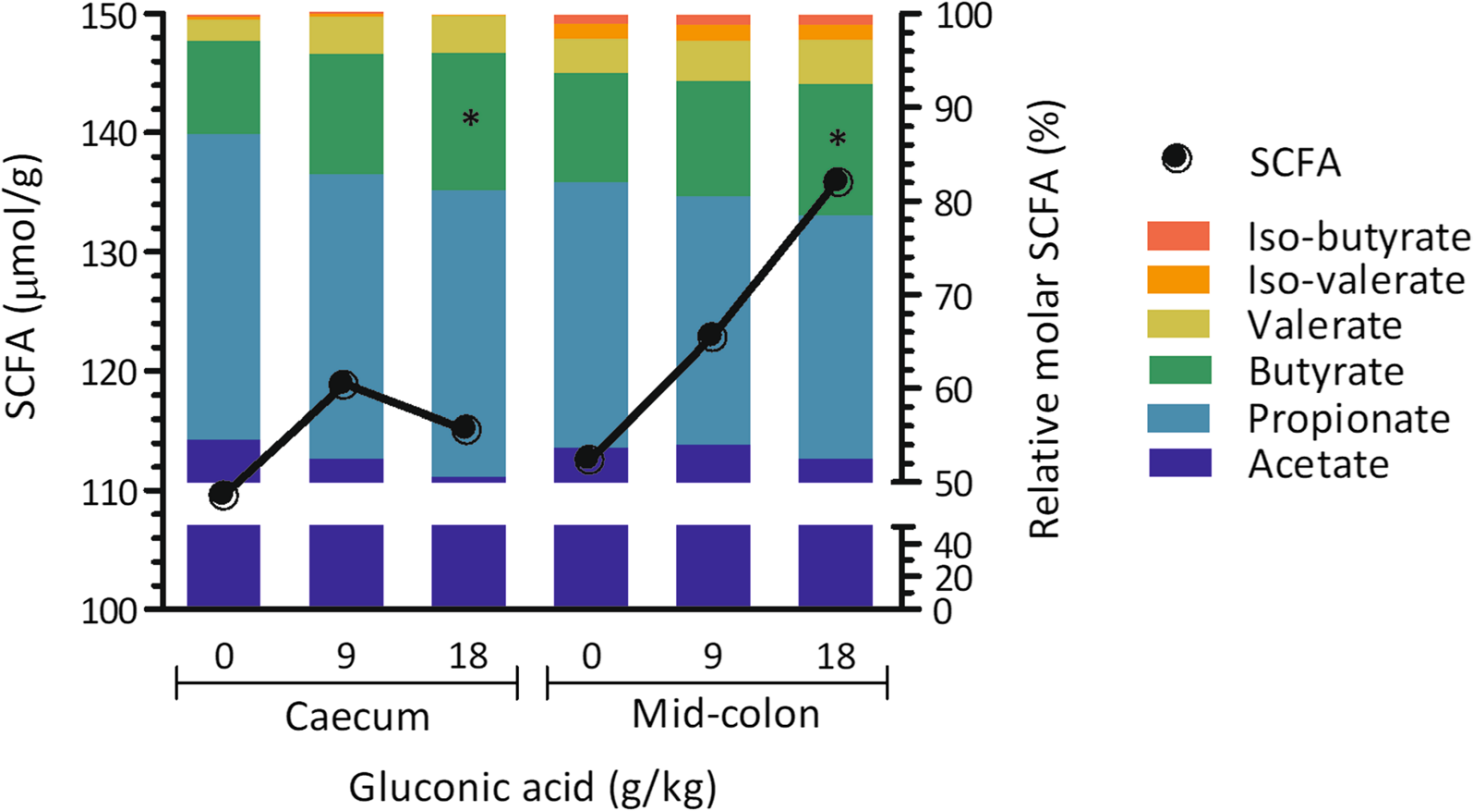
Effect of diet on total (left axis) and relative molar percentages (right axis) of short-chain fatty acids (SCFA) in piglets fed the experimental diets. Piglets were sampled on d 21 (n = 8). *, denotes different relative percentage of butyrate as compared to control in respective section of gastrointestinal tract, *P* < 0.05

**Fig. 2:**
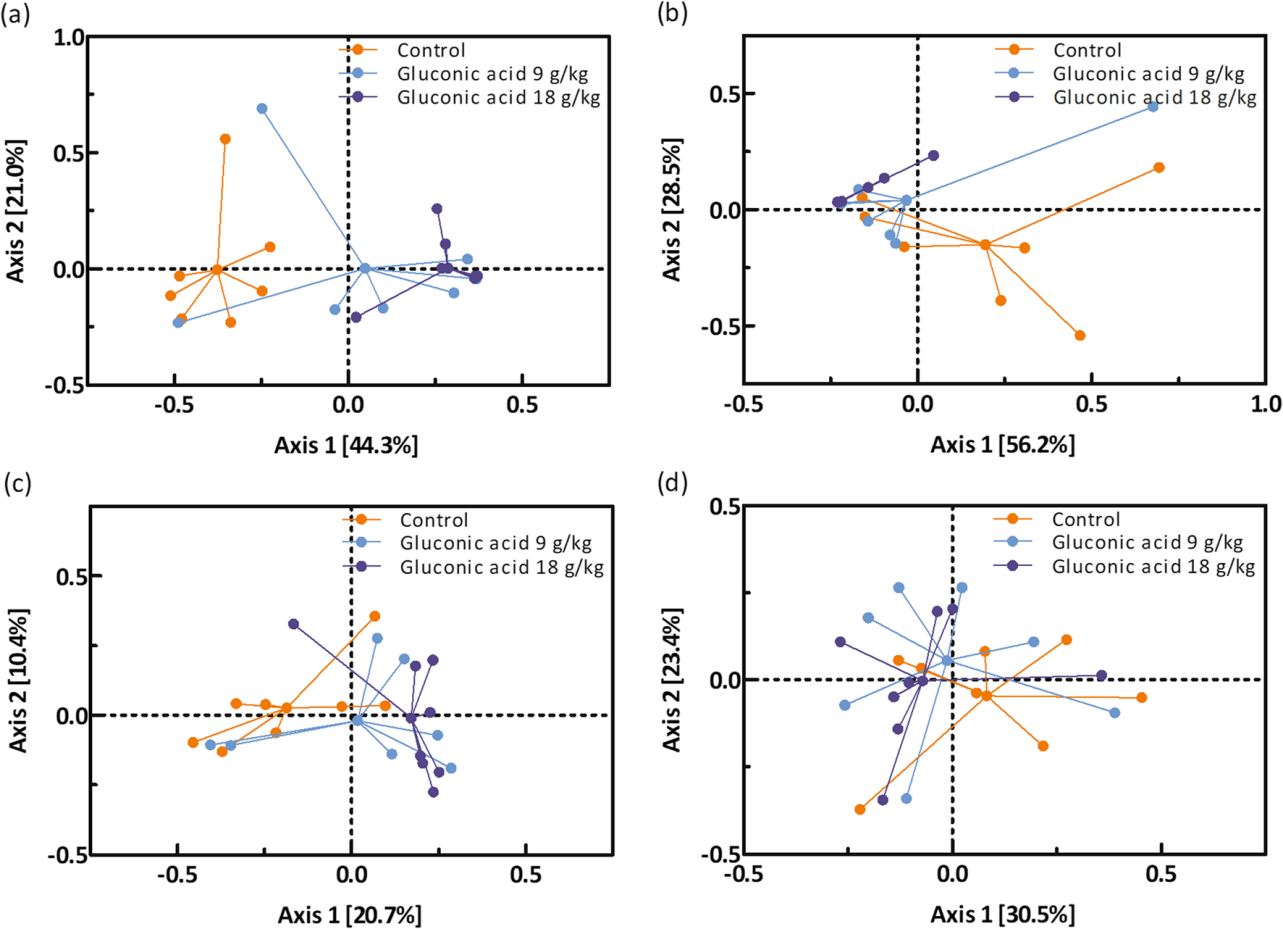
Effect of diet on bacterial community composition presented as PCoA plots based on Bray–Curtis distance in piglets fed the experimental diets. Piglets were sampled on d 21 (n = 8). In distal small intestine at amplicon sequence variant (ASV) (**a**) and genus (**b**) level and in mid-colon at ASV (**c**) and genus (**d**) level. Control diet, orange; gluconic acid at 9 g/kg, light blue; and gluconic acid at 18 g/kg, dark blue. In PCoA plot a, b, and c, bacterial community composition of gluconic acid at 18 g/kg was different from control (all *P* < 0.05); no other differences were observed

**Fig. 3:**
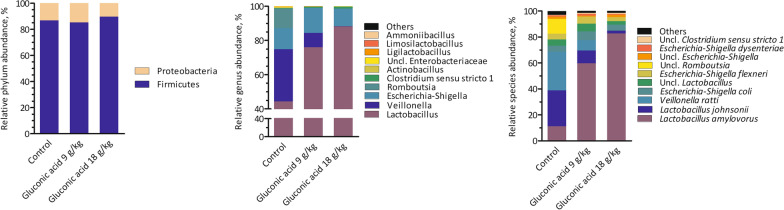
Effect of diet on relative abundance of bacterial taxa in distal small intestine in piglets fed the experimental diets. Piglets were sampled on d 21 (n = 8). All taxa are given at phylum level, 10 most abundant for other taxa. If not classified at respective taxa level, lowest reliable depth of taxonomy is given

**Fig. 4:**
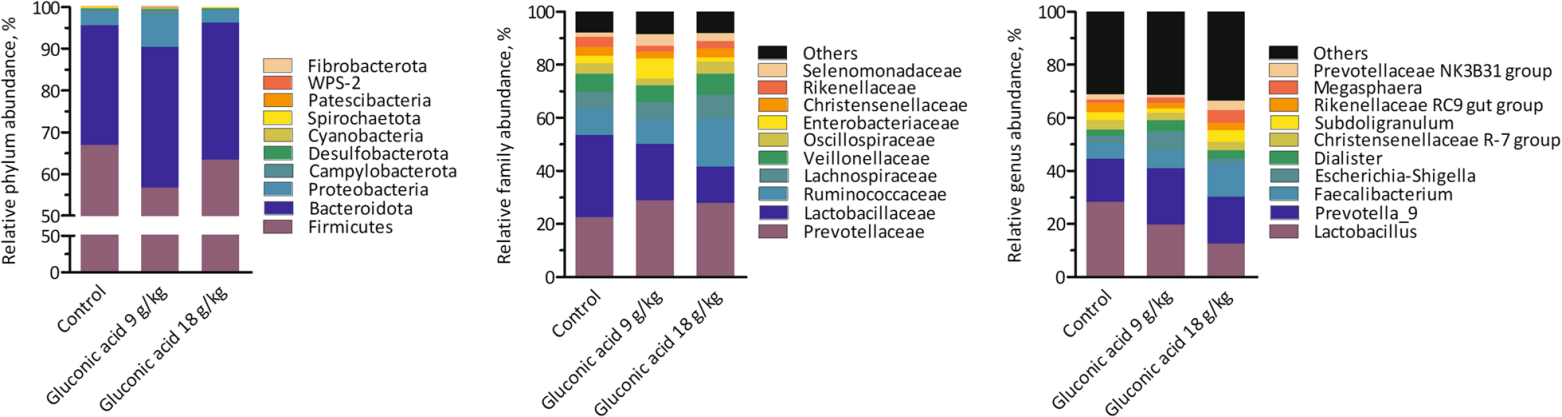
Effect of diet on relative abundance of bacterial taxa in mid-colon in piglets fed the experimental diets. Piglets were sampled on d 21 (n = 8). All taxa are given at phylum level, 10 most abundant for other taxa. If not classified at respective taxa level, lowest reliable depth of taxonomy is given

**Fig. 5:**
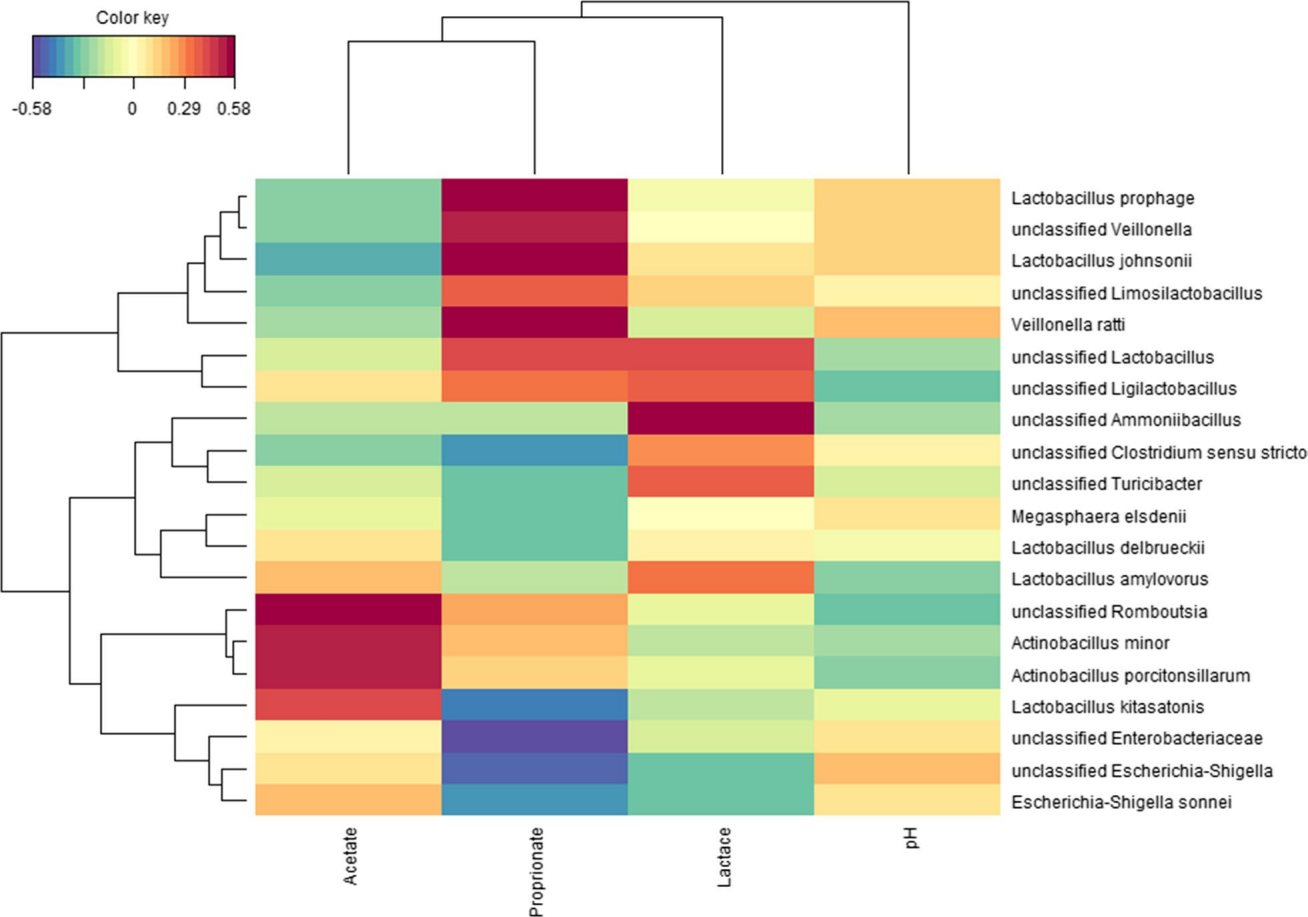
Relationship between bacterial species and pH and fermentation products (absolute concentrations) in samples of distal small intestine in piglets fed the experimental diets. Piglets were sampled on d 21 (N = 24). The heatmap was based on the regularized canonical correlation analysis between relative bacterial abundances at species level and markers of microbial activity

**Fig. 6:**
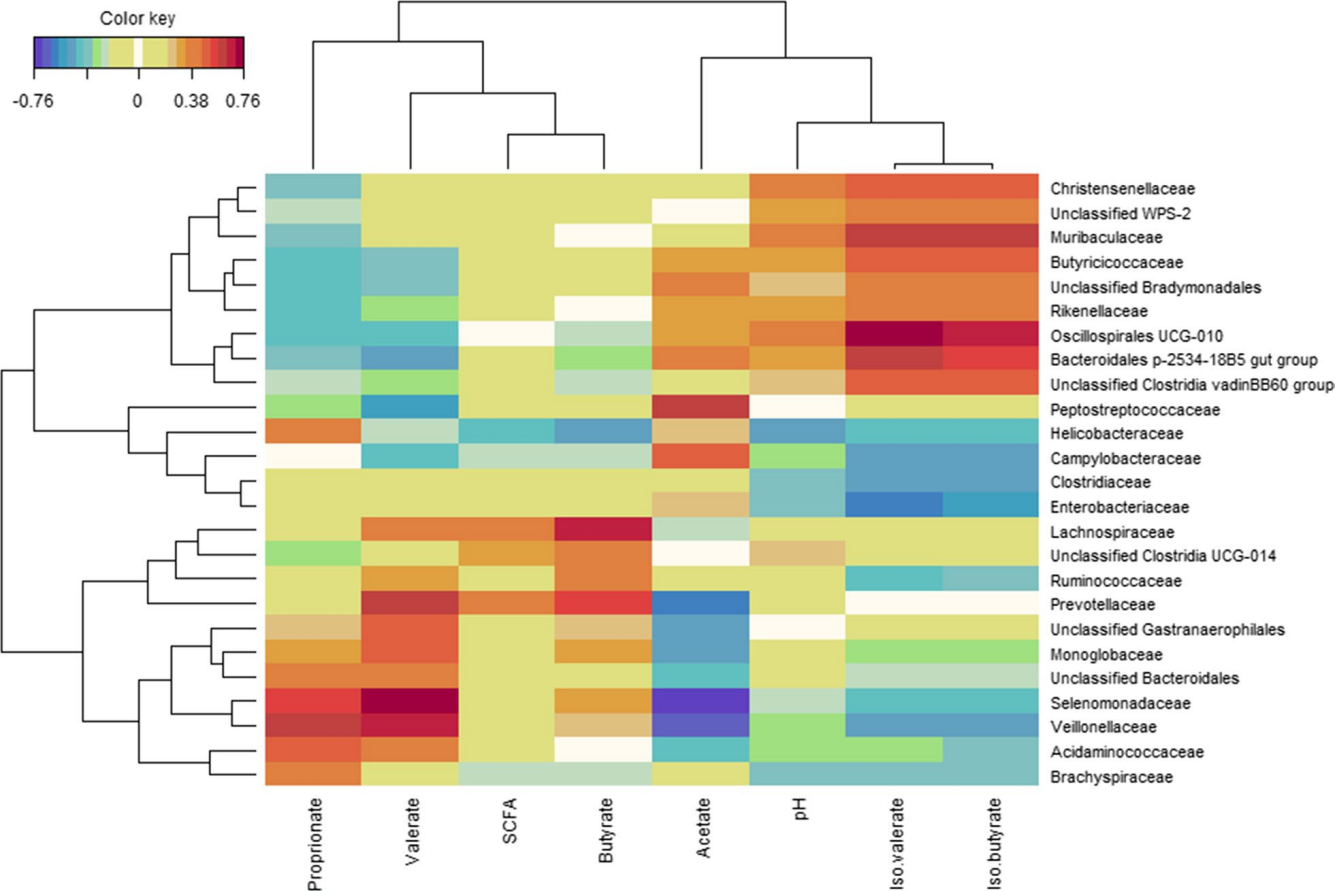
Relationship between bacterial families and pH and fermentation products (total short-chain fatty acids as absolute concentration; acetate, propionate, butyrate, valerate, iso-butyrate, and iso-valerate as relative molar percentages) in samples of mid-colon in piglets fed the experimental diets. Piglets were sampled on d 21 (N = 24). The heatmap was based on the regularized canonical correlation analysis between relative bacterial abundances at family level and markers of microbial activity

Few parameters that characterise digesta in the gastrointestinal tract of piglets sampled on d 21 were found significant (Table 4, Fig. 1). A sharp reduction in pH of gastric contents for group gluconic acid at 18 g/kg was seen: 2.5 *versus* 3.7 and 3.4 in control and gluconic acid at 9 g/kg, respectively (*P* = 0.050) (Table 4). Concentrations of bacterial metabolites in distal small intestine were not affected by treatment. In contrast, in caecal (*P* = 0.046) and mid-colonic (*P* = 0.041) contents an increase in relative butyrate percentage was found (Fig. 1). The percentage butyrate was increased by 4.7 and 2.4% points in caecal and mid-colonic contents in piglets fed 18 g/kg gluconic acid, respectively, as compared to control (*P* < 0.05). A trend suggesting dose-dependent reductions of counts of *Escherichia coli* in digesta of distal small intestine, nearly approaching 1 log_10_/g by gluconic acid at 18 g/kg as compared to control, was seen (*P* = 0.064). Counts of other bacterial groups including the *Lactobacilli* and *Streptococci* in distal small intestine were not affected by treatment. Metagenomic analysis of distal small intestinal and mid-colonic contents was carried out. The bacterial community composition of gluconic acid at 18 g/kg was markedly different from control, both in distal small intestinal and mid-colonic contents at amplicon sequence variant (ASV) level (*P* < 0.05), with clear separation visualised in principal coordinate analysis (PCoA) plots (Fig. 2ac). At genus level, this was only the case in distal small intestine (*P* < 0.05) (Fig. 2b). At ASV level, segregation was obtained along Axis 1, representing most of the variance, whereas at genus level in distal small intestine Axis 2 also contributed to separate treatments. Furthermore, supplementation of gluconic acid reduced indices of alpha diversity in distal small intestine (Table 5). Richness (Chao1) at genus level and diversity (reciprocal Simpson) at ASV level were lowered with highest level of gluconic acid as compared to control (*P* < 0.05), while a trend was found for evenness (Shannon index; *P* = 0.068, at ASV level; *P* = 0.079, at genus level). Albeit it suggests that by feeding gluconic acid at 18 g/kg the number of taxa decreases concomitant with higher dominance of fewer taxa. Similar effects were absent in mid-colon. Most abundant taxa are presented in Figs. 3 and 4, and statistical inferences in relative abundances between treatments are given in Additional files 3 and 4. In distal small intestine, only representatives of Firmicutes (overall relative abundance of 87.4%) and Proteobacteria (12.6%) were found. Interestingly, increased dietary gluconic acid resulted in higher presence of Lactobacillaceae and *Lactobacillus* (*P* < 0.05), and this at the expense of mainly Veillonellaceae and genera *Veillonella* and *Limosilactobacillus* (belonging to Lactobacillaceae) (*P* < 0.05). Other differences at family and genus did not reach significance, though some were notably appealing. Peptostreptococcaceae and its only genus *Romboutsia* were obviously decreased by gluconic acid. A clear shift in presence of members of the Lactobacillaceae family can be observed at species level. *Lactobacillus amylovorus* increased dramatically, i.e., from 11.3 to 82.6% for control and gluconic acid 18 g/kg, respectively (*P* < 0.05), along with higher presence of *Lactobacillus kitasatonis* (overall relative abundance of 0.29%, *P* > 0.05) and *Lactobacillus delbrueckii* (overall relative abundance of 0.07%, *P* > 0.05). Conversely, other species were oppressed like *Lactobacillus johnsonii* (27.5 to 2.1% for control and gluconic acid 18 g/kg, respectively; *P* < 0.05), *Lactobacillus prophage* (*P* < 0.05), and *unclassified Limosilactobacillus* (*P* < 0.05). The decrease in *Veillonella* is a result of lower abundance of the species *Veillonella ratti* (*P* < 0.05) and *unclassified Veillonella* (*P* < 0.05). Ten phyla were determined in mid-colon, with Firmicutes (overall relative abundance of 62.6%), Bacteroidota (31.6%), and Proteobacteria (4.6%) covering 98.8% of all reads. Between and within the 5 most abundant families, i.e., Prevotellaceae (overall relative abundance of 26.3%; Bacteriodota) and Lactobacillaceae, Ruminococcaceae, Lachnospiraceae, and Veillonellaceae (22.1, 12.6, 7.2, and 7.1%, respectively; Firmicutes) alterations took place. At family level it appears that with higher gluconic acid dosage Prevotellaceae, Ruminococcaceae, Lachnospiraceae, and Veillonellaceae increased whereas Lactobacillaceae decreased, however this was not significant. Regarding Prevotellaceae various species showed higher abundance to different extent, with only *unclassified Alloprevotella* being significant (*P* < 0.05). The genera *Faecalibacterium* and *Subdoligranulum* were the main representatives of the Ruminococcaceae, but only numerical sharp increases with the highest level of gluconic acid were notable (*Faecalibacterium prausnitzii*, *unclassified Faecalibacterium*, and *unclassified Subdoligranulum*, *P* > 0.05). The Lachnospiraceae presented a diverse group with amongst them the genera *Roseburia*, *unclassified Lachnospiraceae*, *Oribacterium*, *Agathobacter*, and *[Eubacterium] ruminantium group*. *Agathobacter* was more abundant in pigs fed gluconic acid at 18 g/kg (1.11%) than in control pigs (0.41%) (*P* < 0.05). Remarkable changes within Veillonellaceae occured. Genera *Dialister* (4.19%, *Dialister succinatiphilus* and *unclassified Dialister*, *P* > 0.05), *Megasphaera* (4.66%, mostly *Megasphaera elsdenii*, *P* < 0.05), and *Veillonella* (3.41%, mostly *V. ratti*, *P* < 0.05) showed highest abundance in gluconic acid 9 g/kg, gluconic acid 18 g/kg, and control, respectively. As found in distal small intestine, relative abundances of species within Lactobacillaceae appeared to change by feeding gluconic acid, e.g., *L. johnsonii* and *L. amylovorus* are decreasing and increasing, respectively (both *P* < 0.05), although their abundance is far lower as in distal small intestine and here with the latter species less present than the former. In addition, *L. prophage* and *unclassified Lactobacillaceae HT002* were decreased by gluconic acid 18 g/kg as compared to control (*P* < 0.05). Regularized canonical correlation analysis (rCCA) suggests clear correlations between pH and bacterial metabolites and bacterial species in distal small intestine (Fig. 5). Species that were favoured by gluconic acid can be positively associated with lactate (e.g., *L. amylovorus*), where species that were lowered showed high correlation with propionate (e.g., *L. johnsonii*). High acetate producers were *unclassified Romboutsia* and *Actinobacillus* spp., those were only found in significant numbers in the control treatment. Some members of the Enterobacteriaceae were highly negatively correlated with propionate. Several families within the phylum Bacteriodota (e.g., Muribaculaceae, Rikenellaceae, and Bacteriodales p-2534- 18B5 gut group) were associated with the production of branched-chain fatty acids in mid-colon (Fig. 6). Also, some families within the order of Clostridia did so (Butyricoccaceae and unclassified Clostridia vadinBB60 group), while many other members of this order correlated with butyrate (Lachnospiraceae, unclassified Clostridia UCG-014, and Ruminococcaceae). Prevotellaceae showed high positive relation with both butyrate and valerate, but highly negative with acetate. Other families such as Veillonellaceae and Selenomonadaceae concur with both propionate and valerate and are opposed to acetate levels. Additional file 5 highlights these associations at genus level. Genera that were promoted by gluconic acid associate highly with valerate and/or butyrate (e.g., *Agathobacter*, *Roseburia*, *Faecalibacterium*, *Oribacterium*, *Megasphaera*, and *Dialister*). Contrary, *Lactobacillaceae HT002* and *Veillonella* showed highly negative correlations with these metabolites, they were notably suppressed by feeding gluconic acid.

**Table 4:** Effect of diet on characteristics of digesta in piglets fed the experimental diets (n = 8)^a^

Item	Gluconic acid (g/kg)			SEM	*P*
	0	9	18		
*Stomach*					
pH	3.7	3.5	2.5	0.2	0.05
Dry matter (g/kg)	238	270	237	1	0.248
*Proximal small intestine*					
pH	4.9	5	4.9	0.1	0.991
Dry matter (g/kg)	103	107	91	5	0.473
*Distal small intestine*					
pH	6.3	6.6	6.4	0.1	0.594
Dry matter (g/kg)	98	90	92	6	0.866
*E. coli* (log_10_ CFU^b^/g)	6.4	5.8	5.5	0.2	0.064
Streptococci (log_10_ CFU/g)	7.3	7.3	7.4	0.1	0.87
Lactobacilli (log_10_ CFU/g)	7.5	7.5	7.8	0.1	0.525
Total anaerobes (log_10_ CFU/g)	7	6.8	7.2	0.1	0.385
Acetate (μmol/g)	4.6	3.6	4.7	0.3	0.396
Propionate (μmol/g)	1.1	0.5	0.4	0.2	0.138
Lactate (μmol/g)	39.9	35.9	42.5	5.7	0.914
*Caecum*					
pH	5.3	5.5	5.4	0.1	0.477
Dry matter (g/kg)	105	121	110	5	0.438
*Mid-colon*					
pH	5.8	5.8	5.9	0.1	0.632
Dry matter (g/kg)	182	200	199	10	0.732

^a^Piglets were sampled on d 21

^b^*CFU* colony forming units

**Table 5:** Effect of diet on alpha diversity in piglets fed the experimental diets (n = 8)^abcd^

Item	Gluconic acid (g/kg)			SEM	*P*
	0	9	18		
***Distal small intestine***					
*ASV level*					
Chao1	68.3	55.8	39.7	5.8	0.120
Shannon	2.26	1.64	1.18	0.20	0.068
Reciprocal Simpson	6.95^a^	3.26^ab^	2.22^b^	0.80	0.031
*Genus level*					
Chao1	6.57^a^	5.00^ab^	4.00^b^	0.43	0.040
Shannon	0.58	0.32	0.28	0.06	0.079
Reciprocal Simpson	1.51	1.25	1.28	0.07	0.259
***Mid-colon***					
*ASV level*					
Chao1	386	369	370	26	0.959
Shannon	4.58	4.73	5.05	0.13	0.305
Reciprocal Simpson	44.7	49.8	80.3	8.3	0.160
*Genus level*					
Chao1	60.3	54.5	56.9	3.0	0.748
Shannon	2.56	2.47	2.79	0.12	0.557
Reciprocal Simpson	8.39	7.50	10.80	1.14	0.498

^a^Piglets were sampled on d 21

^b^ASV, amplicon sequence variant

^c^Chao1 index, richness; Shannon index, evenness; reciprocal Simpson index, diversity

^d^Means within row without common superscript are significantly different, P < 0.05

### Barrier f unction, histo-morphology, and gene expression of distal small intestine

**Fig. 7:**
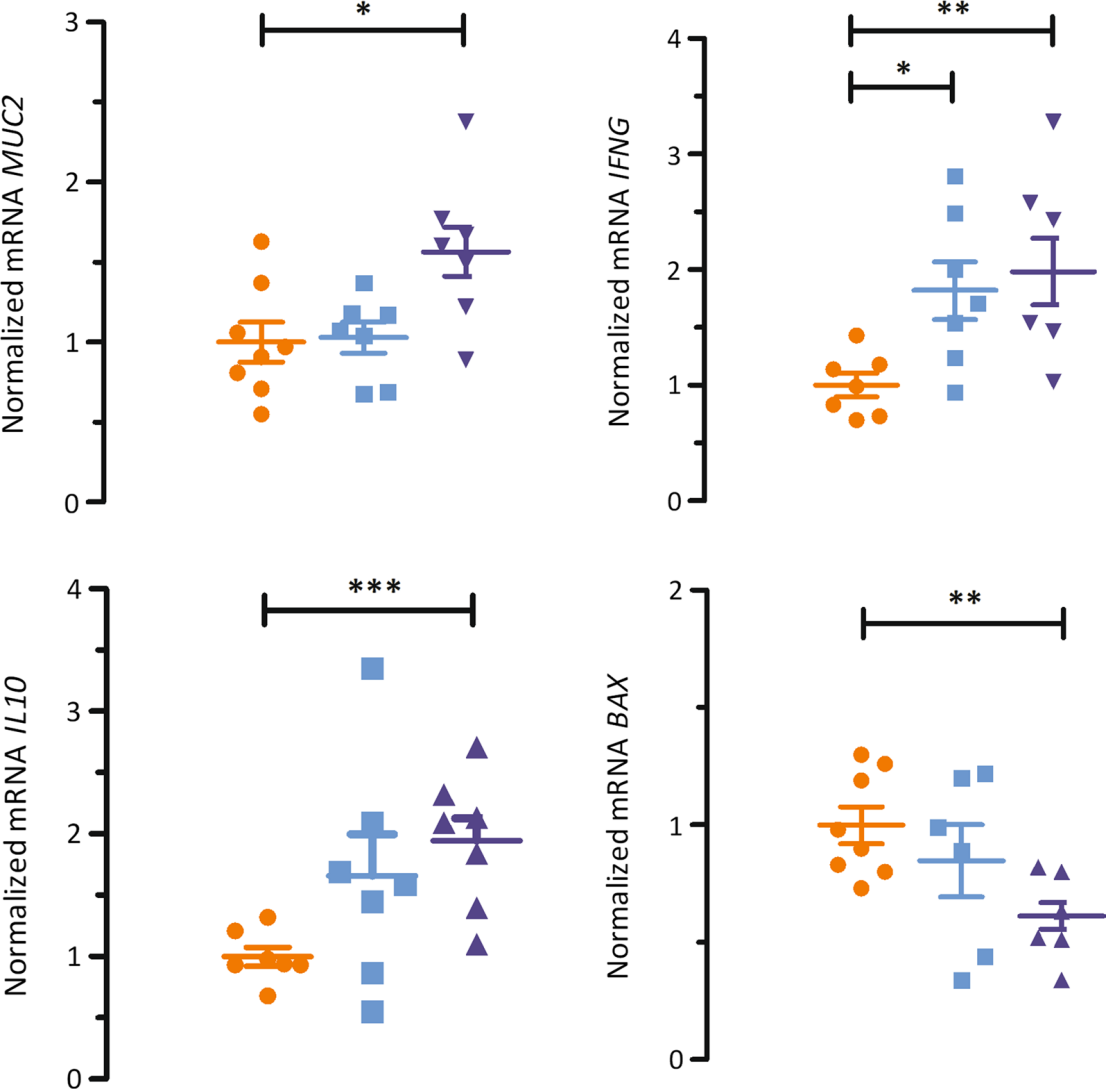
Effect of diet on normalized mRNA in distal small intestinal mucosa of piglets fed the experimental diets. Piglets were sampled on d 21 (n = 8). Control diet, orange; gluconic acid at 9 g/kg, light blue; and gluconic acid at 18 g/kg, dark blue. *, different from control, *P* < 0.05; ** different from control, *P* < 0.001; *** different from control, *P* < 0.0001. *MUC2*: mucin 2; *IFNG*: interferon gamma; *IL10*: interleukin 10; *BAX*: Bcl2 associated x

Neither the apparent permeation coefficient for FD-4 (Papp), nor any histo-morphological measurements or the number of intra-epithelial lymphocytes (IEL) differed among treatments (Table 6). From the 13 genes explored, 4 showed treatment effects (P < 0.05) (Fig. 7). Transcripts of *MUC2*, *IFNG*, and *IL10* were upregulated by highest dose of gluconic acid as compared to control, whereas the lower dose only upregulated *IFNG.* Regarding *IFNG* and *IL10*, expression in piglets with highest gluconic acid dose was nearly twofold as compared to control. The apoptotic activator *BAX* was downregulated by gluconic acid 18 g/kg *versus* control.

**Table 6:** Effect of diet on histo-morphological indices in distal small intestine in piglets fed the experimental diets (n = 8)^ab^

Item	Gluconic acid (g/kg)			SEM	*P*
	0	9	18		
Papp FITC 4 k-Da (log_10_ cm/s.10^–7^)	5.0	4.5	5.2	0.6	0.908
Villus height (μm)	294	287	296	10	0.937
Villus width (μm)	137	136	140	3	0.807
VSA (mm^2^)	0.127	0.122	0.13	0.005	0.846
Crypt depth (μm)	242	247	246	9	0.975
V/C	1.29	1.25	1.32	0.07	0.932
IEL (#10^–3^/μm^2^)	0.329	0.323	0.33	0.018	0.988

^a^Piglets were sampled on d 21

^b^Papp: apparent permeation coefficient; VSA: villus surface area; V/C: villus-crypt ratio; IEL: intra-epithelial lymphocytes

## Discussion

### Piglet performances increase by higher feed intake and improved feed utilization when fed gluconic acid

In the current study, two levels of gluconic acid in the diet of weaned piglets were tested. In the pre-starter period, growth was improved because of both higher feed intake and better feed utilization. Gluconic acid stimulated appetite in the immediate postweaning period, and obviously these responses for feed consumption were higher for gluconic acid at 9 g/kg than at 18 g/kg. In line with this, Biagi et al. [6] found a concave quadratic effect on growth and feed intake over the gluconic acid dose range 0 to 12 g/kg. Poeikhampha and Bunchasak [10] demonstrated that increasing sodium gluconate in the diet of pigs from 0 to 5 g/kg linearly improved BW, ADG, and F:G. Together, it suggests that an optimal dosage to boost performances may exist within the dose range we have tested. Literature data indicate that organic acids may have different effects on feed intake related to the type of organic acid, its chemical and physical form, dosage, age of piglets, and basal diet composition. Excessive amounts of some organic acids may deteriorate feed consumption due to strong odour and flavour and disturbances in acid–base balance [11]. Concerning the latter, uptake of large amounts of acids that are not metabolized in the body may cause metabolic acidosis. Yet, gluconic was confined a mild acid taste [12] and since it is poorly absorbed [4] it may not affect the animal’s acid–base balance. Digestibility coefficients were only and largely affected by the highest dosage of gluconic acid in the current study. The higher apparent ileal organic matter digestibility (+ 0.044) was mainly caused by higher digestibility of the crude protein fraction (+ 0.037). The higher acidity of the gastric contents in piglets fed 18 g/kg gluconic acid could have stimulated proteolytic activity. Indeed, the pH observed in gastric contents of control pigs was 3.7, consistent with Dierick et al. [13] and Michiels et al. [14], whereas it was 2.5 when fed gluconic acid at 18 g/kg. Effects of organic acids on gastric pH have been inconsistent, though in some reports comparable high level of acids showed to have significant impact on acidity of gastric contents [11]. The improvement in apparent ileal crude protein digestibility by 18 g/kg gluconic acid (+ 0.037 or + 3.7% units) corroborates with figures reported by for example Gabert et al. [15] (4.1%, non-significant) and Gabert and Sauer [16] (4.1%, significant) when formic acid (10 g/kg) and fumaric acid (30 g/kg), respectively, were supplemented to a high buffering semi-purified fishmeal diet or wheat-soybean meal diet. Organic acids may also improve the absorption of minerals such as Ca and P by increasing their solubility. Though this hypothesis was not tested here, proof for such an effect of salts of gluconic acid was demonstrated in rats [17] and broiler chickens [18–20]. Organic acids are also expected to slow down the proliferation and/or colonization of undesirable and more pH-sensitive bacteria such as *E. coli* in the stomach and the small intestine. Indeed, a trend was observed for a dose-dependent decrease in *E. coli* numbers in the distal small intestine. The reduction as compared to control was 0.9 log_10_ CFU/g for gluconic acid at 18 g/kg. Counts of other bacterial groups including the lactic acid producing *Lactobacilli* and *Streptococci*, nor the metabolites thereof in distal small intestine were affected by treatment, which was also observed by Biagi et al. [6].

### Gluconic acid affected microbiome and fermentation in gastrointestinal tract

In a rat model, Asano et al. [4] could confirm that gluconic acid is poorly absorbed in the gastrointestinal tract. The fact that pronounced alterations in microbiome composition and fermentation pathways occurred in distal small intestine, caecum and mid-colon may underscore its poor absorption and hence fermentation. To note, the cyclic compound glucuronic acid, a product of oxidation at the 6-carbon of gluconic acid and important constituent of certain natural polysaccharides, was also shown to be partly absorbed and fermented to lactic acid in the pig’s foregut [21]. In our study, beta-diversity shows deviating microbial composition in distal small intestine and mid-colon with gluconic acid at 1.8 g/kg, while the lower dose was clearly intermediate. Regarding the distal small intestine, most striking was the clear shift in presence of the Lactobacillaceae family members. *L. amylovorus* increased substantially, i.e., from 11.3 to 82.6% relative abundance for control and gluconic acid at 18 g/kg, respectively, along with higher abundance of *L. kitasatonis* and *L. delbrueckii*, and lower abundance of *L. johnsonii* and *L. prophage*. As the plating assays demonstrate that the number of viable *Lactobacilli* did not differ across treatments in distal small intestine, congruent to Biagi et al. [6], gluconic acid must have caused a shift within these notable lactic acid producers in favour of *L. amylovorus* and others within a competitive environment like the small intestinal lumen, even though it was clearly shown that many *Lactobacilli* as single strains easily consume gluconic acid albeit to a different degree [5]. In fact, species that were favoured by gluconic acid were positively associated with lactate, where species that were lowered showed high correlation with propionate. At the same time gluconic acid reduced the relative abundance of the G- lactic acid consuming *V. ratti* and *unclassified Veillonella*, both correlated with propionate as demonstrated in the rCCA. It may point to the fact that phosphoenolpyruvate is primarily converted to lactate and accumulates rather than following the succinate pathway and thus giving propionate when feeding gluconic acid [22]. Then, it could be thought that more lactate and less propionate should be recovered in distal small intestinal contents when gluconic acid is supplemented. On the contrary, lactate and propionate levels in this section of the gastrointestinal tract were not significantly different among treatments, though numerically propionate was lowest and lactate highest in treatment gluconic acid at 18 g/kg. The mid-colonic microbiome found in our study, with dominance of Prevotellaceae, Lactobacillaceae, Ruminococcaceae, Lachnospiraceae, and Veillonnellaceae, clearly demonstrates the shift of the postweaning hindgut microbiome to plant-derived glycan metabolism and cross-feeding ecology as reviewed by De Vries and Schmidt [23] and Metzler-Zebeli [24]. Tsukahara et al. [5] showed that the lactate and acetate that were produced from gluconic acid were converted to butyrate by acid-utilizing bacteria, such as *M. elsdenii* and *M. multacida*. Lactate can be used to produce acetyl-CoA, which can be further metabolized in the crotonyl pathway with final conversion of butyryl-CoA by butyrul-CoA:acetate CoA-transferase into butyrate. This conversion utilizes exogenously derived acetate and generates butyrate and acetyl-CoA. The rCCA for mid-colon in our study showed that the genera *Megasphaera* and *Mitsuokella* were positively correlated with butyrate and even more prominent with valerate. Interestingly, while for *Megasphaera* the correlation with butyrate was 0.37 it was markedly higher for valerate, i.e., 0.67. Indeed, Yoshikawa et al. [25] showed that various *M. elsdenii* isolates from pig faeces in Japan produced valerate as predominant SCFA. Tsukahara et al. [26] also found that the addition of lactic acid bacteria and *M. elsdenii* to the medium stimulated the production of butyrate by 60% and of valerate by 50%. Butyrate is essentially synthesized by the condensation of two molecules of acetyl-CoA, and valerate is formed by the condensation of an acetyl-CoA and a propionyl-CoA molecule. It is fair to state that in our study butyrate and/or valerate producers were favoured by feeding gluconic acid. In fact, as both butyrate and valerate were dose-dependently increased in both caecal and mid-colonic contents, it is appealing to report the sum of their relative concentrations, i.e., 12.3, 16.9, and 18.7% in caecum and 15.4, 16.7, and 18.8% in mid-colon for control, gluconic acid at 9 g/kg, and gluconic acid at 18 g/kg, respectively. In caecum, in our study the butyrate concentration reached 16.7 μmol/L when pigs were fed with 18 g/kg gluconic acid for 3 weeks, whereas Tsukahara et al. [5] report concentrations up to 30.0 μmol/kg with 5 g/kg gluconic acid in the diet after 35 d in pigs. Gluconic acid, therefore, seems not only to stimulate butyrate production but also valerate production. However, in the in vitro porcine caecal fermentations of Biagi et al. [6] butyrate was dramatically increased whereas valerate was only marginally elevated. Tran et al. [7] found that gluconic acid yielded the highest butyrate molar ratio among several carbohydrates (29.4%) with a particular reduction of propionate. In contrast, Poeikhampha and Bunchasak [10] found higher accumulation of both propionate and butyrate in caecum by feeding sodium gluconate up to 5 g/kg. It underlines that gluconic acid is ultimately fermented to butyrate, and likely also valerate, thus beneficially altering fermentation patterns in the gastro-intestinal tract, but that the outcome may depend on cross-feeding events between bacterial species.

### Butyrate production may elicit anti-inflammatory responses and steer small intestinal turnover and anabolic metabolism

Next to the well acknowledged physiological effects of butyrate [8, 9], recently, valeric acid glyceride esters were found to promote broiler performance and reduce the incidence of necrotic enteritis in a challenge model [27]. Seeing the increased production of butyrate and valerate by feeding gluconic acid, various effects on gut and peripheral tissues may be expected. Butyrate is the preferred energy-providing substrate for caecal-colonic cells, and in both colonocytes and enterocytes, it may increase epithelial cell proliferation, differentiation, and maturation, decrease apoptosis, and improve barrier function mediated through its influence on gene expression and protein synthesis [9]. Valerate can equally contribute to ATP generation in epithelial cells [28]. Effects of hindgut-produced SCFA on small intestinal tissue are presumably indirectly through neurohormonal mechanisms, for example by the glucagon-like peptide-2 pathway in piglets [29]. Indeed, in our study the pro-apoptotic gene *BAX* was downregulated in distal small intestinal mucosa by gluconic acid at 18 g/kg. The latter is in line with findings by Lacorn et al. [30] who showed that dietary butyrate in pigs led to a reduction in apoptosis in the ileal mucosa without effect on cell mitosis. Further, butyrate is known to exert anti-inflammatory properties [31]. The highest dose of gluconic acid upregulated the transcript of *IFNG* and *IL10*, whereas the lower dose upregulated *IFNG*. Piglets fed gluconic acid at 18 g/kg showed lower number of total white blood cells, caused by particularly lower numbers of lymphocytes in line with in vitro studies [32], while a trend was found for lower numbers of neutrophils in gluconic fed groups. This could indicate lower infection levels in piglets fed the high level of gluconic acid though no further differentiation of these cell populations was undertaken, yet in line with the upregulation of the anti-inflammatory cytokine *IL10*. The latter was also demonstrated by Seamann et al. [33]. Then, equivocal are results on the pro-inflammatory cytokine *IFNG* as our results are opposed to Chevassus et al. [34]. Nonetheless, these authors also found proof for increased secretion of the anti-inflammatory *IL10*, like our results. Transcript of *MUC2* was upregulated by highest dose of gluconic acid. In vitro, butyrate increased dose-dependently the expression of the *MUC2* gene, again supporting the idea that current findings might be mediated by hindgut butyrate production [35]. Since *Lactobacilli* may express to various degrees different cell surface proteins that can adhere to the intestinal mucus layer [36], it may be appealing to hypothesize that current changes in composition within *Lactobacilli* could alter mucus ecology, and potentially barrier function, and thus also be responsible for alterations in the expression of the *MUC2* gene. The fact that we applied high dosages of gluconic acid as precursor of SCFA might be the reason for the very outspoken effects on gene expression of distal small intestinal tissue which are not consistently found in literature studying a wide range of butyrate-based products in livestock.

Among the blood biochemical indices, highly reduced plasma urea was found for both groups fed gluconic acid. This is likely associated with the prebiotic effect of gluconic acid, i.e., providing energy from a carbohydrate source that stimulates bacterial growth consequently consuming ammonia as nitrogen source in distal small intestine and hindgut, like outcomes of other highly fermentable prebiotics such as inulin [37]. We have not measured ammonia in intestinal contents, however, feeding 2.5 g/kg sodium gluconate reduced ammonia levels in caecum of pigs [38] and in the in vitro caecal incubation model, Biagi et al. [6] demonstrated a reduction of ammonia by gluconic acid. The lower plasma urea corroborates inherently with the lower water-to-feed ratio in pre-starter period by feeding gluconic acid as elevated urea provokes higher urinary water excretion. Another point that can be added is that likely a higher ratio between anabolic:catabolic status in animal’s muscle tissues might have prevailed, as creatinine levels are also (not significantly) lower and thus lower deamination of circulatory amino acids, which in turn might be caused by the trophic effects of butyrate on muscle protein metabolism [39].

## Conclusions

Feeding newly weaned piglets with 9 and 18 g/kg gluconic acid in the diet enhanced performance in the immediate postweaning period, though the high dose did not show benefits over the low dose. A dose-dependent increasing prebiotic effect by gluconic acid was evidenced by alterations in relative abundance of lactic acid producing and acid-utilizing bacteria, resulting in increased hindgut butyrate production. Elevated mRNA anti-inflammatory cytokine and survival signalling levels in distal small intestinal mucosa were found by feeding gluconic acid which might be mediated by butyrate. Gluconic acid may have potential to alleviate the postweaning growth-check in pigs.

## Methods

### Animals and experimental design

The study was conducted in accordance with the ethical standards and recommendations for accommodation and care of laboratory animals covered by the European Directive 2010/63/EU on the protection of animals used for scientific purposes and the Belgian royal decree KB29.05.13 on the use of animals for experimental studies. A total of 144 weaning (4-week suckling period; Topigs hybrid x Piétrain) piglets with an average weight of 8.2 kg were used in the feeding trial for 42 days. At arrival, piglets were allocated to 24 pens (2.10 m^2^ per pen, with full slatted floor) with 6 piglets each according to gender and body weight, so that each pen had similar average body weight and equal number of both genders. The 3 treatments were replicated in 8 pens each, according to a completely randomized design and were as follows: control diet, i.e., basal diet; gluconic acid at 9 g/kg, with gluconic acid source added to the basal diet at the expense of barley to obtain the intended level, and gluconic acid at 18 g/kg, with gluconic acid source added to the basal diet at the expense of barley to obtain the intended level. Source of gluconic acid was Gluconic Acid L (500 g/kg gluconic acid, 470 g/kg moisture, 25 g/kg reducing sugars: Roquette Frères, France). From d 0 until d 14, a pre-starter diet was given, followed by a starter diet until d 42. Diets were voided of antibiotics, organic acids, apart from gluconic acid in respective treatments, and supra-nutritional levels of Cu and Zn. The starter diet contained a source of 4 mol/L HCl insoluble ash as digestibility marker. The ingredient and formulated nutrient composition of the basal diets are given in Table 7. Experimental diets were prepared starting from basal diets. Feeds were pellets for all rearing phases. The die used was type 30 × 17 mm. Temperature of mash prior to pelleting was 61, 63 and 63 °C for pre-starter diets control, gluconic acid at 9 g/kg, and gluconic acid at 18 g/kg, respectively; and temperature postpelleting was 72, 74 and 69 °C for the same pre-starter diets. Temperature of mash prior to pelleting was 61, 60 and 60 °C and temperature postpelleting was 78, 74 and 73 °C for starter diets. Nutrient analysis of experimental diets [40] confirms equal nutrient contents across (Additional file 6). Control diets contained small amounts of gluconate originating from sodium gluconate, whereas the supplementary levels of gluconic acid in pre-starter diets amounted to 7.5 and 16.6 g/kg for the diets with gluconic acid at 9 and 18 g/kg, respectively. The analysed levels were thus slightly lower than the intended levels. Opposite to that, in starter diets the supplementary levels were exceeding the intended values, i.e., 11.6 and 21.3 g/kg for same diets.

**Table 7:** Ingredient and formulated nutrient composition (g/kg, unless otherwise stated) of basal diets

Item	Pre-starter	Starter
	(d 0–14)	(d 14–42)
*Ingredient*		
Barley	280	300
Wheat	271	280
Corn	150	100
Soybean meal (480 g/kg CP)		145
Toasted full-fat soybeans	100	100
Dextrose	50.0	
Soy protein concentrate (650 g/kg CP)	45.0	
Potato protein	40.0	
Soy oil	21.5	22.0
Limestone	11.0	12.6
Monocalciumphosphate	7.00	6.39
L-lysine HCl	5.70	5.40
Vitamin and mineral premix^a^	5.00	5.00
Sodium chloride	4.60	4.60
Sodium gluconate	3.00	3.00
L-threonine	2.20	2.50
DL-methionine	2.00	1.84
L-valine	1.02	1.25
L-tryptophan	0.98	0.58
Diamol^b^		10.0
Phytase	0.10	0.10
*Nutrient content*^*c*^		
Dry matter	884	879
Net energy (MJ/kg)	10.5	10.1
Crude protein	174	179
Ether extract	56.2	57.4
Lysine	13.2	12.8
SID Lysine	11.8	11.4
SID M + C/SID Lysine	0.60	0.60
SID Threonine/SID Lysine	0.65	0.65
SID Tryptophan/SID Lysine	0.22	0.21
SID Valine/SID Lysine	0.70	0.70
Calcium	6.70	7.40
Digestible phosphorus	3.30	3.30
Phytase activity (FTU/kg)	1000	1000

^a^Premix providing per kg of diet: vitamin A (retinyl acetate), 10,000 IU; vitamin D3 (cholecalciferol), 2000 IU; vitamin E (dl-α-tocopherol acetate), 40 mg; vitamin K3 (menadione), 1.5 mg; vitamin B1 (thiamine), 1.0 mg; vitamin B2 (riboflavin), 4.0 mg; niacin, 30 mg; D-pantothenic acid, 15 mg; vitamin B6 (pyridoxine–HCl), 1.5 mg; vitamin B12 (cyanocobalamin), 20 µg; folic acid, 0.4 mg; biotin, 0.05 mg; choline chloride, 150 mg; Fe (FeSO_4_.H_2_O), 100 mg; Cu (CuSO_4_.5H_2_O), 20 mg; Mn (MnO), 30 mg; Zn (ZnSO_4_.H_2_O), 70 mg; I (KI), 0.7 mg; Se (Na_2_SeO_3_), 0.25 mg

^b^Diamol; source of 4 mol/L HCl insoluble ash

^c^Calculated nutrient content using matrix values provided by EvaPig® (INRA, UMR1348 PEGASE, Le Clos, 35,590 Saint-Gilles, France)

The ambient temperature in the facility was set to 30 °C and 24 h light was prevailing until d 5 postweaning. From d 6 till d 42, the ambient temperature was linearly adjusted to 26 °C with 18L:6D light schedule. Water and pelleted feed were provided ad libitum throughout the experiment. Piglets as well as feeds were weighed and water consumption registered at d 0, d 14, d 28 and d 42. BW (kg), ADG (g/d), ADFI (g/d), F:G (g/g), Average Daily Water Intake (ADWI, mL/d), and W:F (mL/g) were analysed for all periods. In addition, feed intake was registered at d 2, d 5, d 8, and d 11 to monitor immediate postweaning feed consumption increases. The same trained animal caretaker inspected the animals daily for general health, faecal consistency score and diarrhoea incidence. The faecal consistency score was visually assessed on pen level according to the following scoring system: 1 = hard or slightly moist faeces, clearly formed, normal; 2 = moist or soft faeces, but still with a definite form, sticky; and 3 = watery or liquid faeces, unformed, diarrhoea. If faeces of different consistency in a pen were observed, the highest score present was retained as data. The assessment of diarrhoea incidence (expressed as the percentage of total piglets) was done simultaneously by counting the piglets in the pens receiving faecal consistency score of 3 that show clear signs of diarrhoea, i.e., filthy, wet backside and tail, dehydrated, loss of condition and irritation of the skin around the anus. Faecal consistency score and diarrhoea incidence were assessed until d 21. Occasionally piglets showing prolonged and obvious health issues were treated with antibiotics if needed. Amoxicillin as broad-spectrum antibiotic was used for indications such as respiratory problems, swollen joints, unhealed wounds, severe locomotory problems, and general state of runting. Colistin was used to treat prolonged watery and severe diarrhoea. Antibiotic treatments were carefully monitored.

### Sampling

After 21 days of feeding, one piglet from each pen with body weight close to the average body weight in the respective pen was euthanized by electrocution followed by bleeding. Whole blood was drawn for haematological indices (EDTA as anticoagulant), whereas serum was employed for biochemical measurements (centrifugation at 3000 g for 15 min). Next, the whole gastrointestinal tract was removed, and contents were carefully collected from stomach, proximal small intestine (defined as first 3 m of small intestine), distal small intestine (between 3 and 0.5 m anterior to ileo-caecal valve), distal ileum (0.5 m anterior to ileo-caecal valve), caecum and mid-colon. pH measurement of contents was done for all sections except distal ileum. Complete contents of distal ileum were pooled per treatment, stored at −20 °C, freeze dried and used for apparent ileal digestibility determinations. Aliquots from other sections were frozen at −20 °C pending freeze drying for dry matter determination, whilst aliquots of distal small intestinal, caecal and mid-colonic contents destined for determination of SCFA, and lactic acid were acidified with 2% v/v 6 mol/L H_2_SO_4_. Further, a 1 g subsample of distal small intestinal digesta was taken for enumeration of main bacterial groups by plating. Finally, aliquots of distal small intestinal and mid-colonic digesta were snap frozen and stored at −80 °C pending metagenomic analysis. Meanwhile, a first 5 cm section of the small intestine at 3 m anterior to ileo-caecal valve was taken, rinsed with 9 g/L saline and fixated in a 4% formaldehyde solution for histo-morphological analysis. A second segment (20 cm) was obtained at the same location to assess the macromolecular permeability in Ussing chambers. A third segment (20 cm) was used to harvest mucosa by scraping with glass slide, and frozen immediately in liquid nitrogen and stored at −80 °C pending gene expression analysis.

### Analysis

Apparent ileal digestibility of dry matter, organic matter and protein of pooled samples was assessed using the indicator method with 4 mol/L HCl insoluble ash as marker [40]. Dry matter content was determined by oven drying at 103 °C until constant weight (ISO 6496:1999). Crude ash was analysed by incineration at 550 °C for 4 h in a combustion oven (ISO 5984:2002). Total nitrogen (N) content was determined by the Kjeldahl method (ISO 5983-1:2005). Crude protein content was calculated by multiplying total N with 6.25. The marker was determined according to McCarthy et al. [41].

Haematological indices and biochemical measurements (creatinine, glucose, non-esterified fatty acids, total protein, and urea) were assessed using routine methods at the diagnostic laboratory Animal Health Care (DGZ, Torhout, Belgium).

Bacterial metabolites were determined in digesta of distal small intestine, caecum, and mid-colon. SCFA and lactic acid in small intestinal contents were analysed by a GC method described by Missotten et al. [42] and Van Noten et al. [43]. Briefly, 2-ethyl butyric acid was added to the acidified samples as an internal standard. The mixture was extracted with diethyl ether, followed by derivatization with N-tert-butyldimethylsilyl-N-methyltrifluroacetamide and subsequent analysis on GC. SCFA analysis of caecal and mid-colonic samples was performed on GC after extraction with 10% formic acid with ethyl butyric acid as the internal standard, as described by Castro-Montoya et al. [44].

Bacterial counts (viable counts; log_10_ colony-forming units (CFU) /g fresh digesta) in digesta of distal small intestine were obtained using the ring-plate technique [14, 45]. Seven serial tenfold dilutions were made from 1 g of fresh digesta in a sterilized peptone solution (peptone, 1 g/L; agar, 0.4 g/L; NaCl, 8.5 g/L and cysteine, 0.7 g/L) and plated onto selective media for counting following bacterial groups: Lactobacilli (Rogosa Agar, CM0627B, Oxoid, Basingstoke, UK + 0.132% acetic acid; incubated for 48 h at 37 °C, anaerobically), total anaerobic bacteria (Reinforced Clostridial Agar, CM0151B, Oxoid; incubated for 48 h at 37 °C, anaerobically), *E. coli* (Tryptone Bile X-Glucuronide Agar, CM0945B, Oxoid; incubated for 24 h at 37 °C, aerobically), coliform bacteria (Eosin Methylene Blue Agar, CM0069B, Oxoid; incubated for 24 h at 37 °C, aerobically) and Streptococci (Slanetz & Bartley Medium, CM0377B, Oxoid; incubated for 48 h at 37 °C, aerobically). Data were log_10_ transformed prior to statistical testing. The lower limit of detection was 2 log_10_ CFU/g.

For the microbial composition analysis in distal small intestinal and mid-colonic digesta 16S ribosomal RNA (rRNA) profiling was performed. Genomic DNA was extracted using PSP Spin Stool DNA Plus Kit (Invitek, Westburg, Netherlands) according to the manufacturer’s instructions and modified. Next, the 16S polymerase chain reaction (PCR) libraries were generated and processed as described by Cong et al. [46]. The V1‐V3 hypervariable region of the bacterial 16S rRNA was amplified using (5’‐GAGAGTTTGATYMTGGCTCAG‐3’) and (5’‐ACCGCGGCTGCTGGCAC‐3’) as forward and reverse primers, respectively [47]. After purification, amplicons were sequenced on Illumina Miseq platform (Illumina, San Diego, USA) with the V3 chemistry kit (2 × 300 bp). The 16S rRNA gene sequences in this study were deposited in the NCBI Sequence Read Archive (SRA) database with the accession number PRJNA828581. Originally, 2,990,075 raw reads were obtained. Subsequent bioinformatics was run for distal small intestinal and mid-colonic digesta separately. The raw reads were submitted to the DADA2 package (version 1.20.0; [48]) in R (version 3.3.1, http://www.r-project.org). The Divisive Amplicon Denoising Algorithm (DADA) is based on the identification of single nucleotide sequence variants and provides higher resolution [49]. The raw sequences were quality trimmed and filtered, error models were constructed, ASV’s were inferred, and forward and reverse reads were merged, and chimeras were removed following default settings or adjusted. It resulted in 1,021,759 reads (14,237–80,690 per sample) for distal small intestine and 691,523 reads (11,510–80,691 per sample) for mid-colon. The SILVA (release 138.1; [50]; https://www.arb-silva.de/documentation/release-1381/) was used for taxonomy assignment. Taxonomy data and metadata were merged into a phyloseq object applying the Phyloseq package (version 1.36.0; [51]) in R. Low count ASV’s were removed with a threshold of 0.01% and the ASV table was normalized to 10,765 and 7968 reads per distal small intestinal and mid-colonic sample, respectively, by single rarefaction (Additional file 7). A total of 218 ASV’s, condensed into 2 phyla, 8 families, 13 genera, and 26 species, were used in the downstream analysis for distal small intestine. A total of 1395 ASV’s, condensed into 10 phyla, 42 families, 105 genera, and 140 species, were used in the downstream analysis for mid-colon. PCoA plots based on Bray–Curtis distance were employed to visualize differences in bacterial community composition between different treatments at ASV and genus level (beta diversity). The diversity within bacterial communities per treatment (alpha diversity) was assessed with the Chao1 index (richness), Shannon index (evenness) and reciprocal Simpson index (diversity) at ASV and genus level in Phyloseq.

Fixated sections of the small intestine were processed under standard conditions in an automatic tissue processor [14]. Processing consisted of serial dehydration with ethanol, clearing with xylene and impregnation with paraffin wax. One slide was prepared for each piglet and each slide contained 8 sections cut at 4 mm. Slides were stained with haematoxylin–eosin. Next, villus length (V, from tip to base), mid villus width, and crypt depth (C, from base to opening) of at least 15 well-oriented villi and adjacent crypts were measured and the presence of IEL’s in the epithelial lining was quantified using a microscope (Olympus CX41, Aartselaar, Belgium) equipped with a camera and computer with appropriate software (Olympus Cell B software, Aartselaar, Belgium). The ratio V/C and villus surface area (VSA) was calculated for each villus with adjacent crypt. Mean values for V, C, V/C, and VSA for each piglet were calculated and used for statistical evaluation.

The intestinal permeability was assessed using the Ussing chamber technique as previously described by Wang et al. [52] and Van Noten et al. [43]. In brief, the segment of the distal jejunum was rinsed with saline, stripped from its outer muscle layers, slit longitudinally, and mounted into inserts with an exposed tissue area of 1.07 cm^2^. Two replicate chambers were used per pig and tissues were mounted within 15 min *postmortem*. Fluorescein isothiocyanate–dextran 4-kDa (FD-4; Sigma-Aldrich, Bornem, Belgium) was used as macromolecular probe. Chambers were covered with aluminium foil to protect from light. The marker was added to the mucosal side after 20 min of equilibration to obtain final concentrations of 0.8 mg/mL. Samples were taken from the serosal side every 20 min between 40 and 100 min after mounting the segments. The fluorescent signal of FD-4 was captured using excitation filter at λ = 494 nm and emission filter at λ = 521 nm. The apparent permeation coefficient (Papp) was calculated as: Papp (cm/s) = (dc/dt) × V/c_0_/A, where dc/dt is the change in the marker (FD-4) concentration at the serosal side (acceptor) between 40 and 100 min, (µg/mL/s) calculated from the slope of the concentration–time curve, V is the buffer volume in the luminal side (donor) of the compartment (mL), C_0_ is the initial marker concentration in the donor compartment (µg/mL) and A is the exposed tissue surface area (cm^2^).

RT-qPCR (*MUC2, IL6, TNF, IFNG, IL10, TLR4, OCLN, TJP1, CLDN5, BAX, BCL2, NQO1, and GPX2*) was performed according to the MIQE guidelines [53]. In brief, mucosal total RNA was extracted using the Bio-Rad Aurum Total RNA Fatty and Fibrous Tissue Kit (Bio-Rad Laboratories, Inc., Hercules, USA) according to the manufacturer’s instructions, including an on-column DNase I treatment to remove genomic DNA (gDNA). The concentration and purity (OD_260/280_) of RNA were measured with the NanoDrop ND-1000 (NanoDrop Technologies, Thermo Scientific, Wilmington, USA). One μg RNA was analysed by 1% agarose gel electrophoresis to check RNA integrity (28S and 18S rRNA bands). In addition, a minus-RT control PCR was performed using *YWHAZ* as primer to verify the absence of any gDNA contamination. Following this, 1 μg of high-quality DNA-free RNA was reverse transcribed in the 20 μL reverse-transcription reaction with the ImProm-II cDNA synthesis kit (Promega, Madison, USA), containing both oligo dT and random primers. The obtained cDNA was diluted 10 times with molecular grade water and a control PCR using 2 μL cDNA was performed to verify the reverse-transcription reaction. Primers (Additional file 8) used for genes in the study were designed with Primer3Plus [54]. The repeats, the secondary structure and single nucleotide polymorphism in the target sequence were checked with RepeatMarker [55], mfold [56] and dbSNP [57], respectively. All these primer sequences were gene isoform specific as they were designed based on certain exon-exon boundaries of published pig gene sequences corresponding to the accession number. Primers were then purchased from IDT (Integrated DNA Technologies, Leuven, Belgium). The RT-qPCR was carried out on the CFX96 Touch Real-Time PCR Detection System (Bio-Rad Laboratories, Inc.). Briefly, 2 μL cDNA template, 5 μL 2X KAPA SYBR FAST qPCR Kit Master Mix (Kapa Biosystems, Inc., Wilmington, USA), 2 μL molecular grade water, 0.5 μL forward primer and 0.5 μL reverse primer (5 μmol/L each) were added to a total volume of 10 μL. The amplification conditions were as follows: (1) enzyme activation and initial denaturation (95 °C for 3 min); (2) denaturation (95 °C for 20 s) and annealing/extension and data acquisition (annealing temperature depending on primer for 40 s) repeated 40 cycles; and (3) dissociation (melt curve analysis from 70 to 90 °C with 0.5 °C increment every 5 s). The primers used in this study were first optimized by gradient quantitative real-time PCR. A fivefold dilution series (5 points, from 1 to 625 times dilution) of cDNA as a standard curve was included at 3 gradient temperatures to determine PCR amplification efficiency and specificity. The standard curve was also included in each run to determine PCR efficiency. In this study, PCR amplification efficiencies were consistently between 90 and 110%. Gene-specific amplification was verified by agarose gel electrophoresis and melting curve analysis. Efficiency was used to convert the Cq value into raw data. Data were normalized to the geometric mean of reference genes *HPRT* (M value 0.22) and *RPL4* (0.24) and scaled to control treatment according to Hellemans et al [58].

### Statistical analysis

All data were checked for anomalies and outliers. Next, data for each continuous dependent variable were tested for normal distribution (Mann–Whitney test) and homogeneity of variances (Levene’s test) across treatments. It appeared that data for daily diarrhoea incidence were not normally distributed. Hence, data for this endpoint were evaluated with the non-parametric Kruskal–Wallis test, whereas other endpoints were tested with GLM procedures in SAS Enterprise Guide 7 (SAS Institute, Cary, North Carolina, USA). Data were analysed with the following statistical model (one-way ANOVA):

$$Y_{i} = \mu + GA_{i} + \varepsilon_{i}$$

with *Y*_*i*_ the mean value of treatment with μ, the overall mean, the effect of level of gluconic acid (*i* = 0, 9 and 18 g/kg), and ${\varepsilon }_{i}$ is the error term. Initial pen BW was considered as covariate if significant for performance variables. For performance and physiological measurements pen was also considered as experimental unit, the latter since one pig per pen was taken for sampling. Means are given as least square means and were separated using the Tukey post-hoc test. The Chi-square test was used to evaluate whether the total number of antibiotics treated animals per treatment was different, and this was done at the piglet level. Statistical analysis of daily faeces scores was done by the Chi-square test. Differences were considered significant when *P* < 0.05 and as a tendency for significance when *P* < 0.1. For the microbial composition following 16S rRNA amplicon sequencing, statistical analyses were performed in R using the packages Phyloseq and vegan for community analysis (version 2.5.7; [59]). Significant differences in bacterial community composition between different treatments at ASV and genus level (beta diversity) were identified with pairwise permutational MANOVA on Bray–Curtis distance with Bonferroni correction, using the adonis2 function (vegan). Statistical differences in relative bacterial abundances at phylum, family, genus, and species level between treatments were tested by non-parametric Kruskal–Wallis tests, multiplicity was corrected using the Benjamin-Hochberg false discovery rate (FDR, with FDR = 0.05 for 16S rRNA gene profiling) [60]. Regularized Canonical Correlation Analysis (rCCA) was done to highlight correlations between the metabolic and bacterial community composition (at species level for distal small intestine and genus and family level for mid-colon). rCCA was executed using the mixOmics package with the shrinkage method for estimation of penalisation parameters (version 6.16.3, [61]) in R.

## Supplementary Information

**Additional file 1**: Effect of diet on faecal consistency score and diarrhoea incidence in period d 0-21.

**Additional file 2**: Effect of diet on cumulative feed intake in the pre-starter period.

**Additional file 3**: Effect of diet on relative abundance of bacterial taxa in distal small intestine

**Additional file 4**: Effect of diet on relative abundance of bacterial taxa in mid-colon.

**Additional file 5**: Relationship between bacterial genera and pH and fermentation products in samples of mid-colon.

**Additional file 6**: Analysed nutrient composition of experimental diets.

**Additional file 7**: Rarefaction curves of small intestinal and mid-colonic samples.

**Additional file 8**: Primer sequences used for reverse-transcription quantitative real-time PCR.

## Data Availability

The datasets used and/or analysed during the current study are available from the corresponding author on reasonable request. The 16S rRNA gene sequences in this study were deposited in the NCBI Sequence Read Archive (SRA) database with the accession number PRJNA828581.

## Abbreviations

ADFI: Average daily feed intake
ADG: Average daily gain
ADWI: Average daily water intake
ASV: Amplicon sequence variant
BW: Body weight
C: Crypt depth
CFU: Colony-forming units
F:G: Feed-to-gain ratio
FD-4: Fluorescein isothiocyanate–dextran 4-kDa
IEL: Intra-epithelial lymphocytes
SCFA: Short-chain fatty acids
V: Villus height
VSA: Villus surface area
W:F: Water-to-feed ratio
