# Supplementary material for: Gluconic acid improves performance of newly weaned piglets associated with alterations in gut microbiome and fermentation
Source: Porcine Health Manag. 2023 Apr 5;9:10. doi: 10.1186/s40813-023-00305-1 (PMC10074721; doi:10.1186/s40813-023-00305-1)
Supplement: Supplementary file 1 — Additional file 1: Effect of diet on faecal consistency score and diarrhoea incidence in period d 0-21. [file 40813_2023_305_MOESM1_ESM.docx]

Effect of diet on faecal consistency score (a) and diarrhoea incidence (b) in period d 0-21 in piglets fed the experimental pre-starter diets in period d 0-14, and starter diets in period d 14-42) (n=8). The faecal consistency score was visually assessed on pen level according to the following scoring system: 1 = hard or slightly moist faeces, clearly formed, normal; 2 = moist or soft faeces, but still with a definite form, sticky; and 3 = watery or liquid faeces, unformed, diarrhoea. If faeces of different consistency in a pen were observed, the highest score present was retained as data. The assessment of diarrhoea incidence (expressed as the percentage of total piglets) was done simultaneously by counting the piglets in the pens receiving faecal consistency score of 3 that show clear signs of diarrhoea, i.e., filthy, wet backside and tail, dehydrated, loss of condition and irritation of the skin around the anus. *, different from control at P<0.05. Large within variation was found in period d 4 to 8 preventing finding statistical significance. Curves were smoothed to two neighbours.
