# Supplementary material for: Gluconic acid improves performance of newly weaned piglets associated with alterations in gut microbiome and fermentation
Source: Porcine Health Manag. 2023 Apr 5;9:10. doi: 10.1186/s40813-023-00305-1 (PMC10074721; doi:10.1186/s40813-023-00305-1)
Supplement: Supplementary file 2 — Additional file 2: Effect of diet on cumulative feed intake in the pre-starter period. [file 40813_2023_305_MOESM2_ESM.docx]

Effect of diet on cumulative feed intake in the pre-starter period (d 0-14) in piglets fed the experimental diets (n=8). *, cumulative feed intake different from control, P<0.05.
