# Supplementary material for: Gluconic acid improves performance of newly weaned piglets associated with alterations in gut microbiome and fermentation
Source: Porcine Health Manag. 2023 Apr 5;9:10. doi: 10.1186/s40813-023-00305-1 (PMC10074721; doi:10.1186/s40813-023-00305-1)
Supplement: Supplementary file 3 — Additional file 3: Effect of diet on relative abundance of bacterial taxa in distal small intestine [file 40813_2023_305_MOESM3_ESM.docx]

Effect of diet on relative abundance of bacterial taxa in distal small intestine in piglets fed the experimental diets and sampled on d 21 (n=8)^a^. All taxa are given^b^.

| Taxa level | Taxa | Gluconic acid (g/kg) | | |
| --- | --- | --- | --- | --- |
|  |  | 0 | 9 | 18 |
| Phylum | Firmicutes | 86.90 | 85.19 | 89.62 |
|  | Proteobacteria | 13.10 | 14.81 | 10.38 |
| Family | **Lactobacillaceae** | 44.65^b^ | 76.01^ab^ | 88.31^a^ |
|  | Enterobacteriaceae | 12.47 | 14.81 | 10.38 |
|  | **Veillonellaceae** | 30.48^a^ | 8.45^a^ | 0.12^b^ |
|  | Peptostreptococcaceae | 11.42 | 0 | 0.03 |
|  | Clostridiaceae | 0.34 | 0.70 | 1.10 |
|  | Pasteurellaceae | 0.63 | 0 | 0 |
|  | Paenibacillaceae | 0.01 | 0.01 | 0.04 |
|  | Erysipelotrichaceae | 0 | 0.01 | 0.03 |
| Genus | ***Lactobacillus*** | 44.42^b^ | 75.98^b^ | 88.25^a^ |
|  | ***Veillonella*** | 30.42^a^ | 8.42^a^ | 0.10^b^ |
|  | *Escherichia-Shigella* | 12.30 | 14.64 | 10.24 |
|  | *Romboutsia* | 11.42 | 0 | 0.03 |
|  | *Clostridium sensu stricto 1* | 0.34 | 0.70 | 1.10 |
|  | *Actinobacillus* | 0.63 | 0 | 0 |
|  | *Unclassified Enterobacteriaceae* | 0.18 | 0.17 | 0.14 |
|  | *Ligilactobacillus* | 0.16 | 0.02 | 0.06 |
|  | ***Limosilactobacillus*** | 0.07^a^ | 0.02^ab^ | 0^b^ |
|  | *Ammoniibacillus* | 0.01 | 0.01 | 0.04 |
|  | *Dialister* | 0.05 | 0.01 | 0.01 |
|  | *Megasphaera* | 0.01 | 0.02 | 0.02 |
|  | *Turicibacter* | 0 | 0.01 | 0.03 |
| Species | ***Lactobacillus amylovorus*** | 11.30^b^ | 59.80^a^ | 82.64^a^ |
|  | ***Lactobacillus johnsonii*** | 27.51^a^ | 9.76^ab^ | 2.07^b^ |
|  | ***Veillonella ratti*** | 29.76^a^ | 7.90^b^ | 0.09^c^ |
|  | *Escherichia-Shigella coli* | 4.61 | 6.70 | 4.60 |
|  | *unclassified Lactobacillus* | 4.83 | 6.01 | 2.84 |
|  | *Escherichia-Shigella flexneri* | 4.41 | 5.46 | 2.84 |
|  | *unclassified Romboutsia* | 11.39 | 0 | 0.03 |
|  | *unclassified Escherichia-Shigella* | 1.82 | 0.77 | 1.85 |
|  | *Escherichia-Shigella dysenteriae* | 1.01 | 1.51 | 0.58 |
|  | *unclassified Clostridium sensu stricto 1* | 0.34 | 0.70 | 1.10 |
|  | ***unclassified Veillonella*** | 0.66^a^ | 0.52^a^ | 0.003^b^ |
|  | *Escherichia-Shigella sonnei* | 0.44 | 0.20 | 0.36 |
|  | *Lactobacillus kitasatonis* | 0.19 | 0.15 | 0.50 |
|  | ***Lactobacillus prophage*** | 0.55^a^ | 0.24^ab^ | 0.01^b^ |
|  | *Actinobacillus minor* | 0.53 | 0 | 0 |
|  | *unclassified Enterobacteriaceae* | 0.18 | 0.17 | 0.14 |
|  | *unclassified Ligilactobacillus* | 0.16 | 0.02 | 0.06 |
|  | *Lactobacillus delbrueckii* | 0.02 | 0.01 | 0.18 |
|  | *Actinobacillus porcitonsillarum* | 0.10 | 0 | 0 |
|  | ***unclassified Limosilactobacillus*** | 0.07^a^ | 0.02^ab^ | 0^b^ |
|  | *unclassified Ammoniibacillus* | 0.01 | 0.01 | 0.04 |
|  | *Dialister succinatiphilus* | 0.05 | 0.01 | 0.01 |
|  | *Megasphaera elsdenii* | 0.01 | 0.02 | 0.02 |
|  | *unclassified Turicibacter* | 0 | 0.01 | 0.03 |
|  | *Romboutsia ilealis* | 0.03 | 0 | 0 |
|  | *Lactobacillus taiwanensis* | 0.03 | 0 | 0 |

^a^ Means within row without common superscript are significantly different, P<0.05.

^b^ If not classified at respective taxa level, lowest reliable depth of taxonomy is given and denoted as unclassified.
