# Supplementary material for: Gluconic acid improves performance of newly weaned piglets associated with alterations in gut microbiome and fermentation
Source: Porcine Health Manag. 2023 Apr 5;9:10. doi: 10.1186/s40813-023-00305-1 (PMC10074721; doi:10.1186/s40813-023-00305-1)
Supplement: Supplementary file 4 — Additional file 4: Effect of diet on relative abundance of bacterial taxa in mid-colon. [file 40813_2023_305_MOESM4_ESM.docx]

Effect of diet on relative abundance of bacterial taxa in mid-colon in piglets fed the experimental diets and sampled on d21 (n=8)^a^. All taxa are given at pylum level, for other taxa levels groups at overall relative abundance >0.5% are shown^b^.

| Taxa level | Taxa | Gluconic acid (g/kg) | | |
| --- | --- | --- | --- | --- |
|  |  | 0 | 9 | 18 |
| Phylum | Firmicutes | 66.91 | 56.68 | 63.38 |
|  | Bacteroidota | 28.60 | 33.67 | 32.87 |
|  | Proteobacteria | 3.43 | 7.77 | 2.93 |
|  | Campylobacterota | 0.52 | 1.20 | 0.27 |
|  | Desulfobacterota | 0.26 | 0.23 | 0.28 |
|  | Cyanobacteria | 0 | 0.29 | 0.05 |
|  | Spirochaetota | 0.18 | 0.04 | 0.09 |
|  | Patescibacteria | 0.08 | 0.05 | 0.03 |
|  | WPS-2 | 0.02 | 0.03 | 0.06 |
|  | Fibrobacterota | 0.01 | 0.03 | 0.06 |
| Family | Prevotellaceae | 22.54 | 28.76 | 27.80 |
|  | Lactobacillaceae | 30.97 | 21.28 | 13.81 |
|  | Ruminococcaceae | 10.02 | 9.39 | 18.05 |
|  | Lachnospiraceae | 6.00 | 6.46 | 8.92 |
|  | Veillonellaceae | 6.99 | 6.20 | 7.94 |
|  | Oscillospiraceae | 3.92 | 2.66 | 4.69 |
|  | Enterobacteriaceae | 2.85 | 7.54 | 1.47 |
|  | Christensenellaceae | 3.49 | 2.65 | 3.27 |
|  | Rikenellaceae | 3.76 | 2.24 | 2.93 |
|  | Selenomonadaceae | 1.59 | 4.17 | 3.05 |
|  | Muribaculaceae | 1.40 | 1.55 | 1.62 |
|  | [Eubacterium] coprostanoligenes group | 1.11 | 1.20 | 1.36 |
|  | Acidaminococcaceae | 1.23 | 0.70 | 0.85 |
|  | Succinivibrionaceae | 0.53 | 0.23 | 1.43 |
|  | Campylobacteraceae | 0.41 | 1.19 | 0.24 |
| Genus | *Lactobacillus* | 28.27 | 19.75 | 12.56 |
|  | *Prevotella_9* | 16.35 | 21.24 | 17.68 |
|  | *Faecalibacterium* | 5.69 | 6.77 | 12.79 |
|  | *Escherichia-Shigella* | 2.77 | 7.21 | 1.47 |
|  | *Dialister* | 2.38 | 4.19 | 3.26 |
|  | *Christensenellaceae R-7 group* | 3.49 | 2.61 | 3.27 |
|  | *Subdoligranulum* | 3.11 | 1.74 | 4.27 |
|  | *Rikenellaceae RC9 gut group* | 3.64 | 2.14 | 2.85 |
|  | ***Megasphaera*** | 1.18^b^ | 1.90^b^ | 4.66^a^ |
|  | *Prevotellaceae NK3B31 group* | 1.95 | 1.06 | 3.53 |
|  | *Roseburia* | 1.43 | 2.14 | 2.52 |
|  | *Prevotella_7* | 0.90 | 2.87 | 2.01 |
|  | *Oscillospiraceae UCG-002* | 2.21 | 1.27 | 1.81 |
|  | *Prevotella* | 1.17 | 2.04 | 1.66 |
|  | *Unclassified Muribaculaceae* | 1.40 | 1.55 | 1.62 |
|  | *Unclassified Selenomonadaceae* | 0.47 | 2.53 | 1.26 |
|  | *Prevotellaceae UCG-003* | 1.44 | 0.77 | 1.56 |
|  | *Unclassified [Eubacterium] coprostanoligenes group* | 1.11 | 1.20 | 1.36 |
|  | ***Veillonella*** | 3.41^a^ | 0.10^b^ | 0.02^b^ |
|  | *Limosilactobacillus* | 1.55 | 0.85 | 0.83 |
|  | *Oscillospiraceae UCG-005* | 0.62 | 0.61 | 1.87 |
|  | *Mitsuokella* | 0.71 | 1.09 | 0.96 |
|  | *Unclassified Lachnospiraceae* | 0.78 | 0.53 | 1.20 |
|  | *Oribacterium* | 0.67 | 0.82 | 0.88 |
|  | ***Lactobacillaceae HT002*** | 1.14^a^ | 0.68^ab^ | 0.43^b^ |
|  | *Oscillospiraceae NK4A214 group* | 0.83 | 0.53 | 0.73 |
|  | ***Agathobacter*** | 0.41^a^ | 0.57^ab^ | 1.11^b^ |
|  | *Succinivibrio* | 0.52 | 0.11 | 1.32 |
|  | *Acidaminococcus* | 0.94 | 0.57 | 0.37 |
|  | *Lachnospiraceae NK4A136 group* | 0.83 | 0.41 | 0.61 |
|  | *[Eubacterium] ruminantium group* | 0.44 | 0.39 | 0.92 |
|  | *Campylobacter* | 0.41 | 1.19 | 0.24 |
|  | ***Alloprevotella*** | 0.41^b^ | 0.33^b^ | 0.77^a^ |
| Species | *unclassified Prevotella_9* | 16.18 | 20.84 | 17.29 |
|  | ***Lactobacillus johnsonii*** | 23.35^a^ | 12.74^a^ | 1.95^b^ |
|  | *Faecalibacterium prausnitzii* | 3.51 | 3.95 | 7.56 |
|  | ***Lactobacillus amylovorus*** | 1.19^b^ | 4.24^ab^ | 8.83^a^ |
|  | *unclassified Faecalibacterium* | 2.18 | 2.82 | 5.23 |
|  | *unclassified Christensenellaceae R-7 group* | 3.49 | 2.61 | 3.27 |
|  | *unclassified Subdoligranulum* | 3.11 | 1.74 | 4.27 |
|  | *unclassified Rikenellaceae RC9 gut group* | 3.64 | 2.14 | 2.85 |
|  | ***Megasphaera elsdenii*** | 1.04^b^ | 1.47^b^ | 4.15^a^ |
|  | *unclassified Prevotellaceae NK3B31 group* | 1.95 | 1.06 | 3.53 |
|  | *Dialister succinatiphilus* | 1.70 | 2.16 | 2.40 |
|  | *unclassified Prevotella_7* | 0.90 | 2.87 | 2.01 |
|  | *unclassified Oscillospiraceae UCG-002* | 2.21 | 1.27 | 1.81 |
|  | *Escherichia-Shigella coli* | 1.14 | 3.15 | 0.75 |
|  | *unclassified Roseburia* | 1.17 | 1.85 | 1.80 |
|  | *Escherichia-Shigella flexneri* | 1.23 | 3.10 | 0.52 |
|  | *unclassified Muribaculaceae* | 1.40 | 1.55 | 1.62 |
|  | *unclassified Selenomonadaceae* | 0.47 | 2.53 | 1.26 |
|  | *unclassified Prevotella* | 0.85 | 1.82 | 1.44 |
|  | *unclassified Prevotellaceae UCG-003* | 1.44 | 0.77 | 1.56 |
|  | *unclassified [Eubacterium] coprostanoligenes group* | 1.11 | 1.20 | 1.36 |
|  | ***Veillonella ratti*** | 3.27^a^ | 0.10^b^ | 0.02^b^ |
|  | *unclassified Dialister* | 0.68 | 2.03 | 0.86 |
|  | *unclassified Limosilactobacillus* | 1.55 | 0.85 | 0.83 |
|  | *unclassified Oscillospiraceae UCG-005* | 0.62 | 0.61 | 1.87 |
|  | ***Lactobacillus prophage*** | 1.56^a^ | 1.05^ab^ | 0.11^b^ |
|  | *unclassified Lactobacillus* | 1.16 | 0.87 | 0.56 |
|  | *unclassified Lachnospiraceae* | 0.78 | 0.53 | 1.20 |
|  | *unclassified Mitsuokella* | 0.68 | 1.00 | 0.88 |
|  | *unclassified Oribacterium* | 0.67 | 0.82 | 0.88 |
|  | ***unclassified Lactobacillaceae HT002*** | 1.14^a^ | 0.68^ab^ | 0.43^b^ |
|  | *unclassified Oscillospiraceae NK4A214 group* | 0.83 | 0.53 | 0.73 |
|  | ***unclassified Agathobacter*** | 0.41^b^ | 0.57^ab^ | 1.11^a^ |
|  | *unclassified Succinivibrio* | 0.52 | 0.11 | 1.32 |
|  | *unclassified [Eubacterium] ruminantium group* | 0.44 | 0.39 | 0.92 |
|  | ***unclassified Alloprevotella*** | 0.41^b^ | 0.33^b^ | 0.77^a^ |

^a^ Means within row without common superscript are significantly different, P<0.05.

^b^ If not classified at respective taxa level, lowest reliable depth of taxonomy is given and denoted as unclassified.
