# Supplementary material for: Gluconic acid improves performance of newly weaned piglets associated with alterations in gut microbiome and fermentation
Source: Porcine Health Manag. 2023 Apr 5;9:10. doi: 10.1186/s40813-023-00305-1 (PMC10074721; doi:10.1186/s40813-023-00305-1)
Supplement: Supplementary file 6 — Additional file 6: Analysed nutrient composition of experimental diets. [file 40813_2023_305_MOESM6_ESM.docx]

Analysed nutrient composition (g/kg) of experimental diets for the pre-starter (d 0 to 14) and starter (d 14 to 42) phase.

| Item | | Gluconic acid (g/kg) | | | | | |
| --- | --- | --- | --- | --- | --- | --- | --- |
|  |  | Pre-starter | | | Starter | | |
|  |  | 0 | 9 | 18 | 0 | 9 | 18 |
| Dry matter |  | 899 | 894 | 888 | 910 | 908 | 904 |
| Ash |  | 34 | 40 | 39 | 51 | 51 | 53 |
| Crude protein, N x 6.25 |  | 177 | 174 | 171 | 183 | 182 | 179 |
| Ether extract |  | 64 | 64 | 65 | 62 | 64 | 65 |
| Sum amino acids |  | 173 | 171 | 166 | 176 | 175 | 176 |
| Lysine |  | 12.0 | 12.3 | 12.2 | 11.6 | 12.5 | 12.5 |
| Methionine |  | 4.9 | 5.2 | 4.7 | 4.5 | 4.5 | 4.4 |
| Cystine |  | 3.2 | 3.0 | 3.1 | 3.5 | 3.1 | 3.1 |
| Threonine |  | 8.9 | 8.6 | 8.3 | 8.1 | 8.6 | 8.5 |
| Tryptophane |  | 3.2 | 3.3 | 3.1 | 2.9 | 3.0 | 2.9 |
| Valine |  | 9.1 | 8.8 | 8.7 | 8.7 | 8.8 | 8.9 |
| Leucine |  | 13.6 | 13.5 | 13.1 | 13.1 | 12.9 | 13.1 |
| Tyrosine |  | 6.2 | 6.2 | 6.2 | 6.0 | 5.9 | 5.9 |
| Phenylalanine |  | 8.5 | 8.4 | 8.2 | 8.6 | 8.4 | 8.5 |
| Histidine |  | 3.8 | 3.8 | 3.7 | 4.2 | 4.1 | 4.2 |
| Gluconate |  | 3.5 | 11.0 | 20.1 | 3.3 | 14.9 | 24. 6 |
