# Supplementary material for: Gluconic acid improves performance of newly weaned piglets associated with alterations in gut microbiome and fermentation
Source: Porcine Health Manag. 2023 Apr 5;9:10. doi: 10.1186/s40813-023-00305-1 (PMC10074721; doi:10.1186/s40813-023-00305-1)

Rarefaction curves of small intestinal (a) and mid-colonic (b) samples. ASV (amplicon sequence variant) table was normalized to 10765 and 7968 reads (sample size) per distal small intestinal and mid-colonic sample, respectively, by single rarefaction.


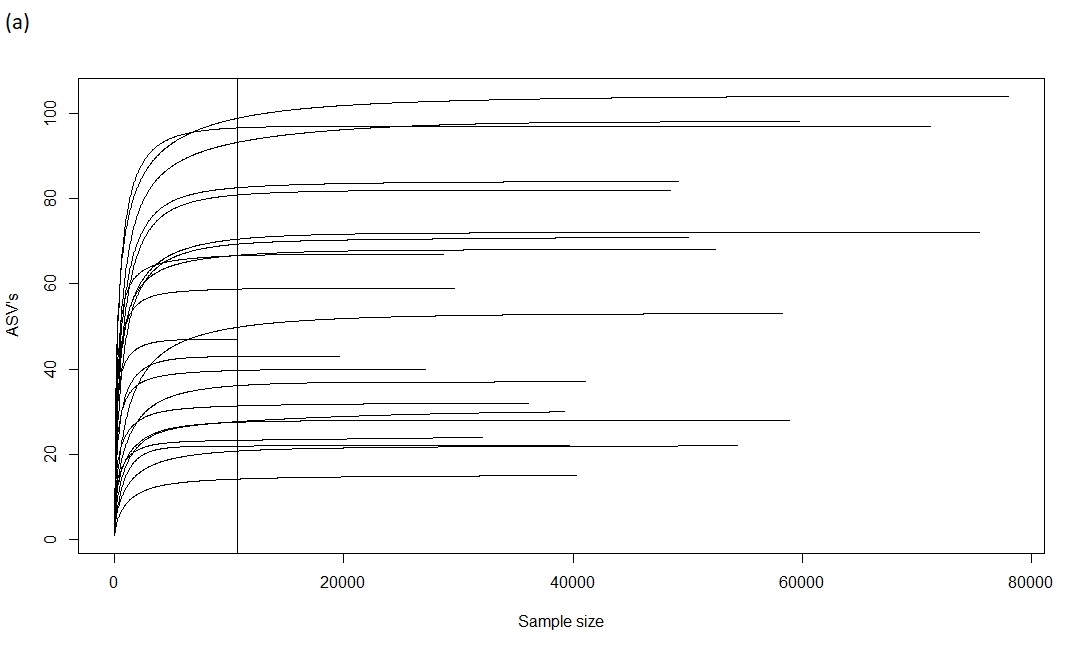


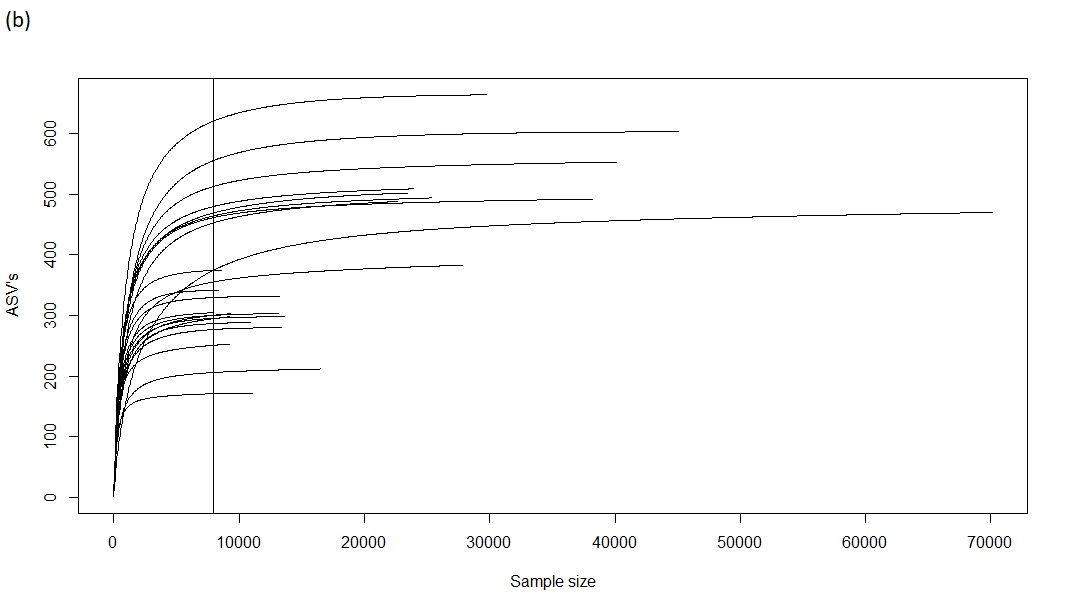

Supplement: Supplementary file 7 — Additional file 7: Rarefaction curves of small intestinal and mid-colonic samples. [file 40813_2023_305_MOESM7_ESM.docx]
