# Supplementary material for: Gluconic acid improves performance of newly weaned piglets associated with alterations in gut microbiome and fermentation
Source: Porcine Health Manag. 2023 Apr 5;9:10. doi: 10.1186/s40813-023-00305-1 (PMC10074721; doi:10.1186/s40813-023-00305-1)
Supplement: Supplementary file 8 — Additional file 8: Primer sequences used for reverse-transcription quantitative real-time PCR. [file 40813_2023_305_MOESM8_ESM.docx]

Primer sequences used for reverse-transcription quantitative real-time PCR.

| Gene symbol^a^ | | Accession number | Nucleotide sequence of primers, 5’-3’ | | Product length (bp) | Ta (°C) |
| --- | --- | --- | --- | --- | --- | --- |
|  |  |  | Forward | Reverse |  |  |
| *MUC2* | | XM_013989745.1 | AGGACGACACCATCTACCTCACTC | GGCCAGCTCGGGAATAGACCTT | 132 | 58 |
| *IL6* | | NM_214399.1 | AGCCCACCAGGAACGAAAGAGAG | GGCAGTAGCCATCACCAGAAGCA | 165 | 58 |
| *TNF* | | NM_214022.1 | CATGATCCGAGACGTGGAGC | AACCTCGAAGTGCAGTAGGC | 151 | 62 |
| *IFNG* | | NM_213948.1 | GCTTTTCAGCTTTGCGTGACT | CACTCTCCTCTTTCCAATTCTTCA | 166 | 58 |
| *IL10* | | XM_013979620.1 | GCGCTGTGTGAGTAGCAGACCA | CAGAGAGAAATGAGCAAGAGACAGGACT | 123 | 62 |
| *TLR4* | | NM_001113039.2 | TTCTTGCAGTGGGTCAAGGA | GACGGCCTCGCTTATCTGAC | 135 | 58 |
| *OCLN* | | NM_001163647.2 | TGGAGAGACGCCCATTCGACCA | CGTGGAGGCAACACAGGCAAAG | 169 | 65 |
| *TJP1* | | XM_003480423.3 | ATCTCGGAAAAGTGCCAGGA | CCCCTCAGAAACCCATACCA | 172 | 61 |
| *CLND5* | | NM_001161636.1 | GTGGTCCGCGAGTTCTACGA | CTTGACAGGGAAGCCGAGGT | 171 | 60 |
| *BAX* | XM_013998624.2 | | TTCCTTCGAGATCGGCTGCTGG | TCTTCCAGATGGTGAGCGAGGC | 142 | 65 |
| *BCL2* | | XM_021099593.1 | AGAGCGTCAACCGGGAGATGT | GCCTTCAGAGACAGCCAGGAGA | 178 | 64 |
| *NQO1* | | [NM_001159613.1](https://www.ncbi.nlm.nih.gov/entrez/viewer.fcgi?db=nucleotide&id=227430402) | AGAGTGGAAGAAACGCCTGG | GTTGTCCGTCGGGATGGATT | 187 | 60 |
| *GPX2* | | [NM_001115136.1](https://www.ncbi.nlm.nih.gov/entrez/viewer.fcgi?db=nucleotide&id=169636442) | AACAGCCTCAAGTACGTCCG | ATGAGGGAAAACGGGTCGTC | 152 | 60 |
| *HPRT1* | | XM_021079504.1 | CCGAGGATTTGGAAAAGGT | CTATTTCTGTTCAGTGCTTTGATGT | 181 | 60 |
| *RPL4* | | XM_005659862.3 | CAAGAGTAACTACAACCTTC | GAACTCTACGATGAATCTTC | 122 | 58 |
| *YWHAZ* | | NM_001315726.1 | TGATGATAAGAAAGGGATTGTGG | GTTCAGCAATGGCTTCATCA | 203 | 60 |

^a^ *MUC2*: mucin 2; *IL6:* interleukin 6; *TNF*: tumor necrosis factor alpha; *IFNG*: interferon gamma; *IL10*: interleukin 10; *TLR4*: toll-like receptor-4; *OCLN*: occludin; *TJP1*: tight junction protein 1; *CLND5*: claudin 5; *BAX*: Bcl2 associated x; *BCL2*: B-cell lymphoma 2; *NQO1*: NAD(P)H quinone dehydrogenase 1; *GPX2*: glutathione peroxidase 2; *HPRT1*: hypoxanthine phosphoribosyltransferase 1; *RPL4*: ribosomal protein L4; *YWHAZ*: tyrosine 3-monooxygenase/tryptophane 5-monooxygenase activation protein zeta
